# HDAC inhibitors enhance the immunotherapy response of melanoma cells

**DOI:** 10.18632/oncotarget.17950

**Published:** 2017-05-17

**Authors:** Laurence Booth, Jane L. Roberts, Andrew Poklepovic, John Kirkwood, Paul Dent

**Affiliations:** ^1^ Department of Biochemistry and Molecular Biology, Virginia Commonwealth University, Richmond, VA 23298-0035, USA; ^2^ Department of Medicine, Virginia Commonwealth University, Richmond, VA 23298-0035, USA; ^3^ University of Pittsburgh Cancer Institute, Melanoma and Skin Cancer Program, Hillman Cancer Research Pavilion Laboratory, Pittsburgh, PA 15232, USA

**Keywords:** melanoma, immunotherapy, HDAC inhibitor, pazopanib, autophagy

## Abstract

We focused on the ability of the pan-histone deacetylase (HDAC) inhibitors AR42 and sodium valproate to alter the immunogenicity of melanoma cells. Treatment of melanoma cells with HDAC inhibitors rapidly reduced the expression of multiple HDAC proteins as well as the levels of PD-L1, PD-L2 and ODC, and increased expression of MHCA. In a cell-specific fashion, melanoma isolates released the immunogenic protein HMGB1 into the extracellular environment. Very similar data were obtained in ovarian and H&NSCC PDX isolates, and in established tumor cell lines from the lung and kidney. Knock down of HDAC1, HDAC3, HDAC8 and HDAC10, but not HDAC6, recapitulated the effects of the HDAC inhibitors on the immunotherapy biomarkers. Using B16 mouse melanoma cells we discovered that pre-treatment with AR42 or sodium valproate enhanced the anti-tumor efficacy of an anti-PD-1 antibody and of an anti-CTLA4 antibody. In the B16 model, both AR42 and sodium valproate enhanced the anti-tumor efficacy of the multi-kinase inhibitor pazopanib. In plasma from animals exposed to [HDAC inhibitor + anti-PD-1], but not [HDAC inhibitor + anti-CTLA4], the levels of CCL2, CCL5, CXCL9 and CXCL2 were increased. The cytokine data from HDAC inhibitor plus anti-PD-1 exposed tumors correlated with increased activated T cell, M1 macrophage, neutrophil and NK cell infiltration. Collectively, our data support the use of pan-HDAC inhibitors in combination with kinase inhibitors or with checkpoint inhibitor antibodies as novel melanoma therapeutic strategies.

## INTRODUCTION

Prior studies have demonstrated that pan-HDAC inhibitors such as AR42 and sodium valproate can combine with multi-kinase inhibitors such as pazopanib to kill tumor cells [1–6]. At present there is a phase I trial at VCU Massey Cancer Center combining pazopanib and the histone deacetylase inhibitor AR42 in sarcoma and renal carcinoma (NCT02795819). An expansion cohort of this trial, at the defined RP2D of the drug combination, is to be recruited from refractory melanoma patients.

Pazopanib, as well as being a kinase inhibitor, is also a potent inhibitor of chaperone ATPase activities and recently it was shown that [pazopanib + AR42] treatment of melanoma cells reduced the abilities of HSP90 and HSP70 to chaperone RAF-1 and B-RAF [7, 8]. Chaperone acetylation has been reported to inhibit chaperone function and it was lately shown that the acetylation of HSP90, HSP70 and GRP78 enhanced the ability of pazopanib to inhibit their chaperone ATPase activities [9–11]. In addition, AR42 or sodium valproate and the [pazopanib + HDAC inhibitor] combination all acted to reduce the protein levels of multiple HDAC proteins arguing for a highly complex mechanism of drug combination action [1, 12, 13].

Immunotherapy has become a first line therapeutic regimen in melanoma. Antibodies that blockade the functions of PD-1, PD-L1 and CTLA-4 have all been approved as melanoma therapeutics within the last 5 years [14, 15]. Histone deacetylase inhibitors are known to increase MHC class I and II expression on the cell surface which would facilitate anti-tumor responses from both the innate and adaptive immune systems. Other studies have linked HDAC inhibitors to both increased and decreased expression of PD-L1 and PD-L2 on tumor cells [16–20]. Recent studies have shown that transient *in vivo* treatment of dabrafenib/trametinib resistant human melanoma tumors growing in athymic mice with AR42 results in a significant increase in animal survival [1]. The tumors under control conditions at nadir contained low levels of macrophages, neutrophils and natural killer cells, whereas AR42 treated tumors at nadir had elevated infiltrated levels of these immune cells. These effects were associated with: reduced plasma levels of metalloproteases 1-3; IL-10; IL-12 family cytokines; reduced IL-6 activity; and with increased G-CSF levels.

The present studies are a continuation of our earlier recent work in melanoma combining the multi-kinase and chaperone inhibitor pazopanib with the pan-histone deacetylase inhibitors AR42 and sodium valproate. In the present manuscript we demonstrate that AR42 and sodium valproate, in multiple tumor types, reduce the expression of PD-L1, PD-L2 and ornithine decarboxylase (ODC) and increase the expression of the class I MHC molecule MHCA. In many tumor isolates AR42 and valproate also promoted the extracellular release of the immunogenic protein HMGB1. *In vivo* AR42 or sodium valproate enhanced the anti-tumor efficacy of anti-PD-1 and of anti-CTLA4 antibodies in the B16 melanoma model. Collectively, the findings within this manuscript strongly argue that the rational coupling of pan-HDAC inhibitors to current immunotherapies could provide expanded response rates and improved outcomes for melanoma patients (and beyond), and that specific HDAC therapies may not be effective due to the overlapping regulatory mechanisms performed by the multitude of HDACs in human tumor cells.

## RESULTS

**Figure 1:**
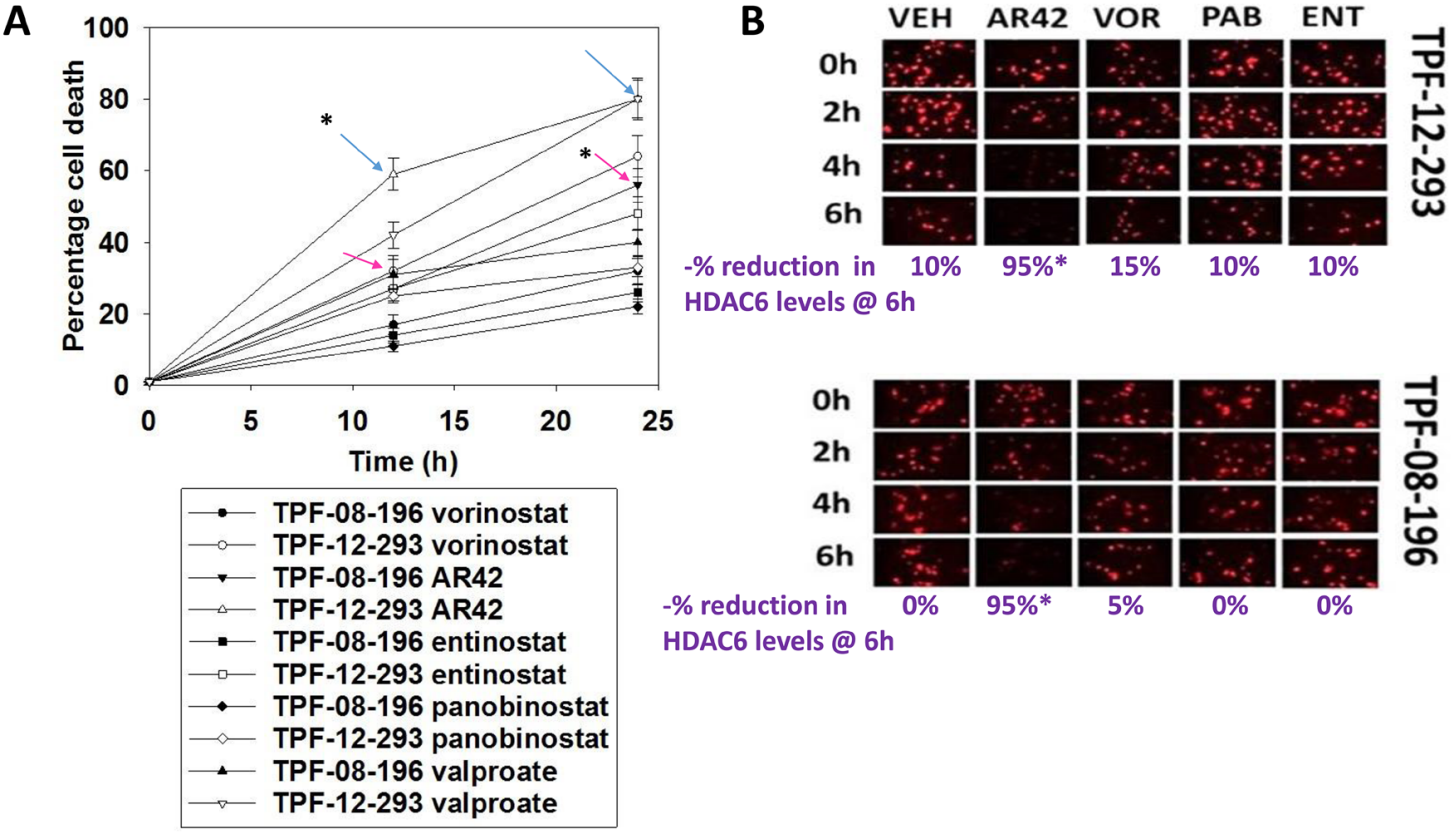
AR42 and sodium valproate at their safe C max concentrations have greater efficacy at killing melanoma cells than vorinostat, panobinostat and entinostat at their C max concentrations **(A)** TPF-08-196 and TPF-12-293 cells were treated with vehicle control, vorinostat (1.5 μM); AR42 (1.4 μM), entinostat (200 nM), panobinostat (50 nM) or sodium valproate (750 μM) for 12h and for 24h. At each time point cells were subjected to live/dead cell viability assays. Green cells = viable; yellow/red cells = dying/dead. (n =3 +/-SEM). Blue arrows indicate AR42 in TPF-12-293 cells and red arrow indicate AR42 in TPF-08-196 cells. # p < 0.05 greater levels of cell killing than under all other conditions. **(B)** TPF-08-196 and TPF-12-293 cells were treated with vehicle control, vorinostat (0.6 μM); AR42 (0.6 μM), entinostat (80 nM), or panobinostat (20 nM) for 6h. Cells were fixed in place and immunostaining performed to detect the expression of HDAC6. (n = 3 +/-SEM). * p < 0.05 less than corresponding staining intensity values under all other conditions.

Our initial studies continued onward from the final data sets examining drug resistance mechanisms in MEL28 tumor cells, as presented in Booth *et al.* [1]. The pan-HDAC inhibitors AR42 and sodium valproate both exhibited greater anti-melanoma killing effects at their safe plasma C max concentrations than did other clinically relevant HDAC inhibitors (Figure 1A). The red arrows in the graph correspond to AR42 lethality against TPF-11-08-196 cells and the blue arrows correspond to AR42 lethality against TPF-12-293 cells. At 40% of their safe plasma C max concentrations, AR42, but not the other HDAC inhibitors, was competent to rapidly reduce the expression of HDAC6. Prior studies had shown that this reduction in HDAC6 levels required autophagosome formation [1].

**Figure 2:**
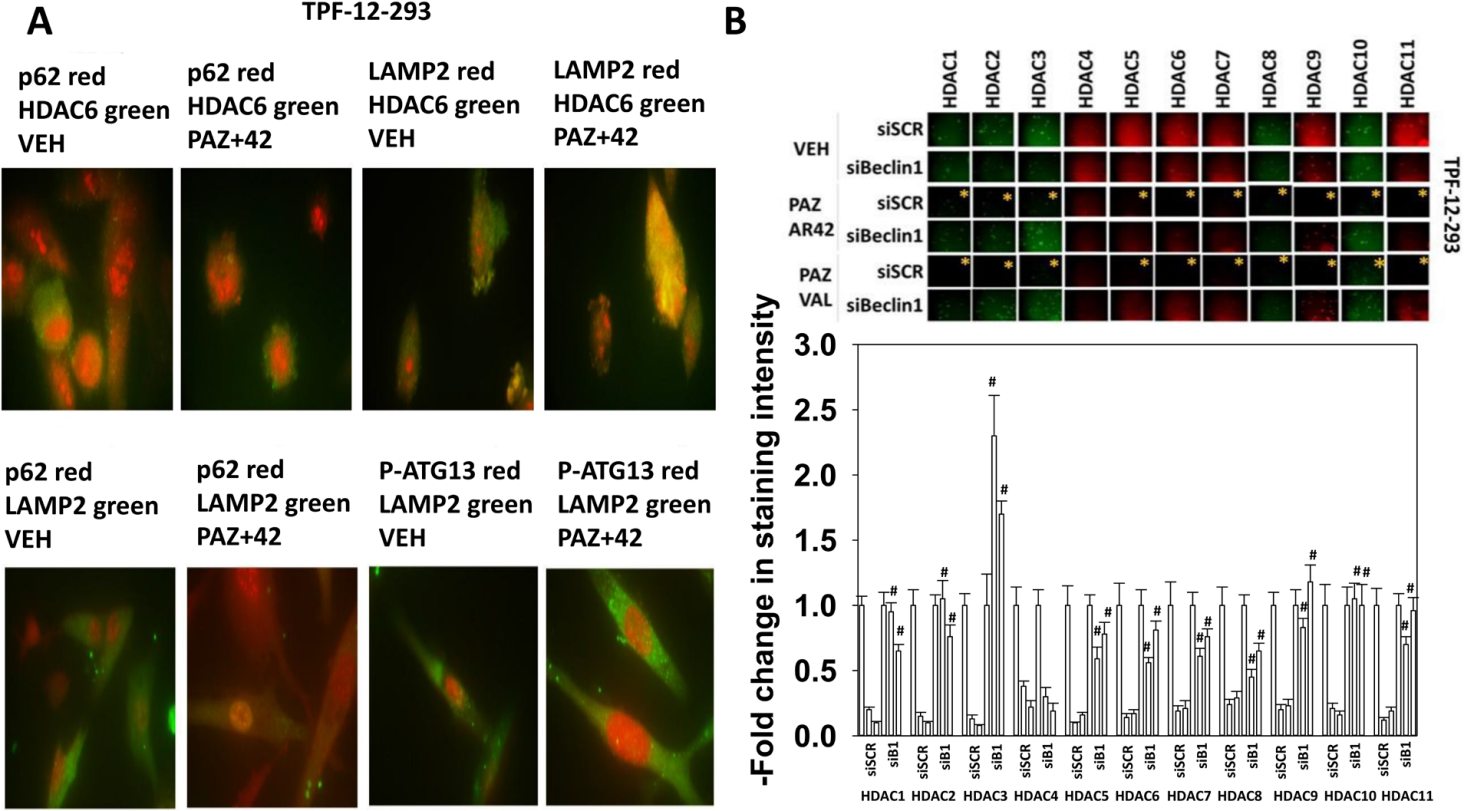
Exposure of melanoma cells to [pazopanib + HDAC inhibitor] reduces the protein levels of HDACs in a Beclin1-dependent fashion **(A)** TPF-12-293 cells were treated with vehicle control or with [pazopanib (1 μM) + AR42 (600 nM)] for 6h. Cells were fixed in place and immunostaining at 60X magnification performed to detect the proteins indicated in each image; HDAC6, p62, LAMP2, and P-ATG13 S318. **(B)** TPF-12-293 cells were transfected with a scrambled control or with an siRNA to knock down Beclin1. Twenty-four h after transfection cells were treated with vehicle control or with [pazopanib (1 μM) + AR42 (600 nM)] or with [pazopanib (1 μM) + valproate (250 μM)] for 6h. Cells were fixed in place and immunostaining performed to detect the expression of HDACs1-11. The bars read from left to right: vehicle control; [PAZ+AR42]; [PAZ+VAL] (n = 3 +/-SEM). * p < 0.05 less than corresponding value in vehicle control treated cells; # p < 0.05 greater than corresponding value in siSCR cells.

Treatment of vemurafenib resistant TPF-12-293 melanoma cells with [pazopanib + AR42] promoted the co-localization of HDAC6 with LAMP2 (Figure 2A). HDAC6 did not co-localize with p62/SQSTM1, p62 weakly co-localized with LAMP2 and phospho-ATG13 S318 did not co-localize with LAMP2. See also data presented regarding p62 and LAMP2 co-localization in ref [21]. Collectively, together with our prior findings that the proteasome inhibitor bortezomib did not block the reduction in HDAC6 levels, this data argues that HDAC6 was being degraded in lysosomes [1]. Based on our data with HDAC6, we then determined whether the reduced expression of other HDACs, seen after [pazopanib + HDAC inhibitor] exposure required “autophagy.” Knock down of Beclin1 prevented [pazopanib + HDAC inhibitor] treatment reducing the expression of HDAC1, HDAC2, HDAC3, HDAC5, HDAC6, HDAC8, HDAC9, HDAC10 and HDAC11 (Figure 2B; Supplementary Figure 1). [Pazopanib + HDAC inhibitor] exposure reduced the expression of HDAC4 that was not rescued by knock down of Beclin1. Collectively, these findings argue that drug combinations which induce autophagosome formation may have the potential to regulate protein acetylation *via* the degradation of HDAC proteins. Furthermore, this implies that HDACs that are weakly inhibited by current HDAC inhibitors, e.g. HDAC5, can be down-regulated through the autophagic degradation process.

**Figure 3:**
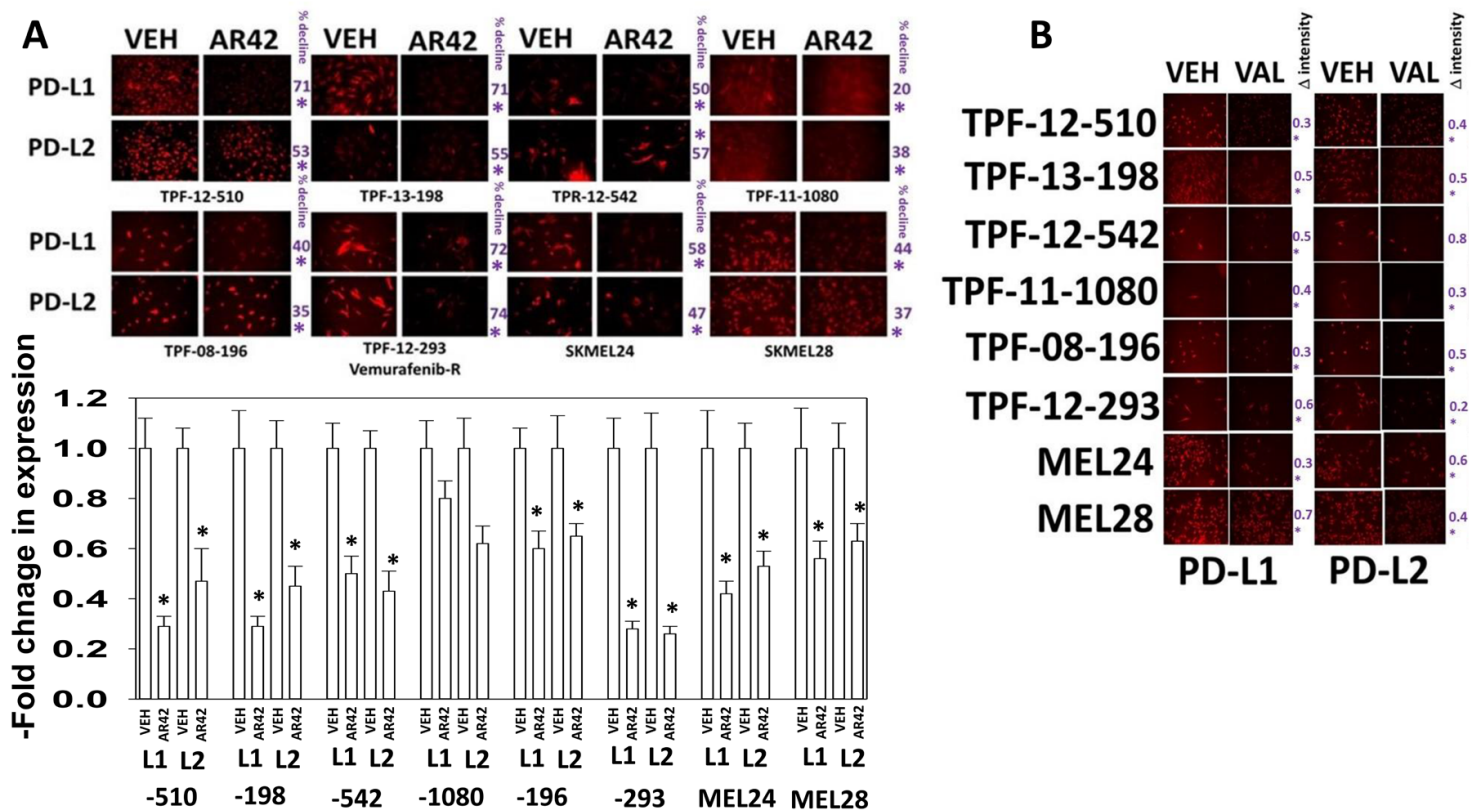
HDAC inhibitors regulate the expression of PD-L1, PD-L2 in melanoma cells **(A)** Melanoma cells were treated with vehicle control or with AR42 (600 nM) for 6h. Cells were fixed in place and immunostaining performed to determine the gross (10X mag.) expression of PD-L1, PD-L2, MHCA and ODC. (n = 3 +/-SEM) *p < 0.05 less than vehicle control. **(B)** Melanoma cells were treated with vehicle control or with sodium valproate (250 μM) for 6h. Cells were fixed in place and immunostaining performed to determine the gross (10X mag.) expression of PD-L1, PD-L2, MHCA and ODC. (n = 3 +/-SEM) *p < 0.05 less than vehicle control.

**Figure 4:**
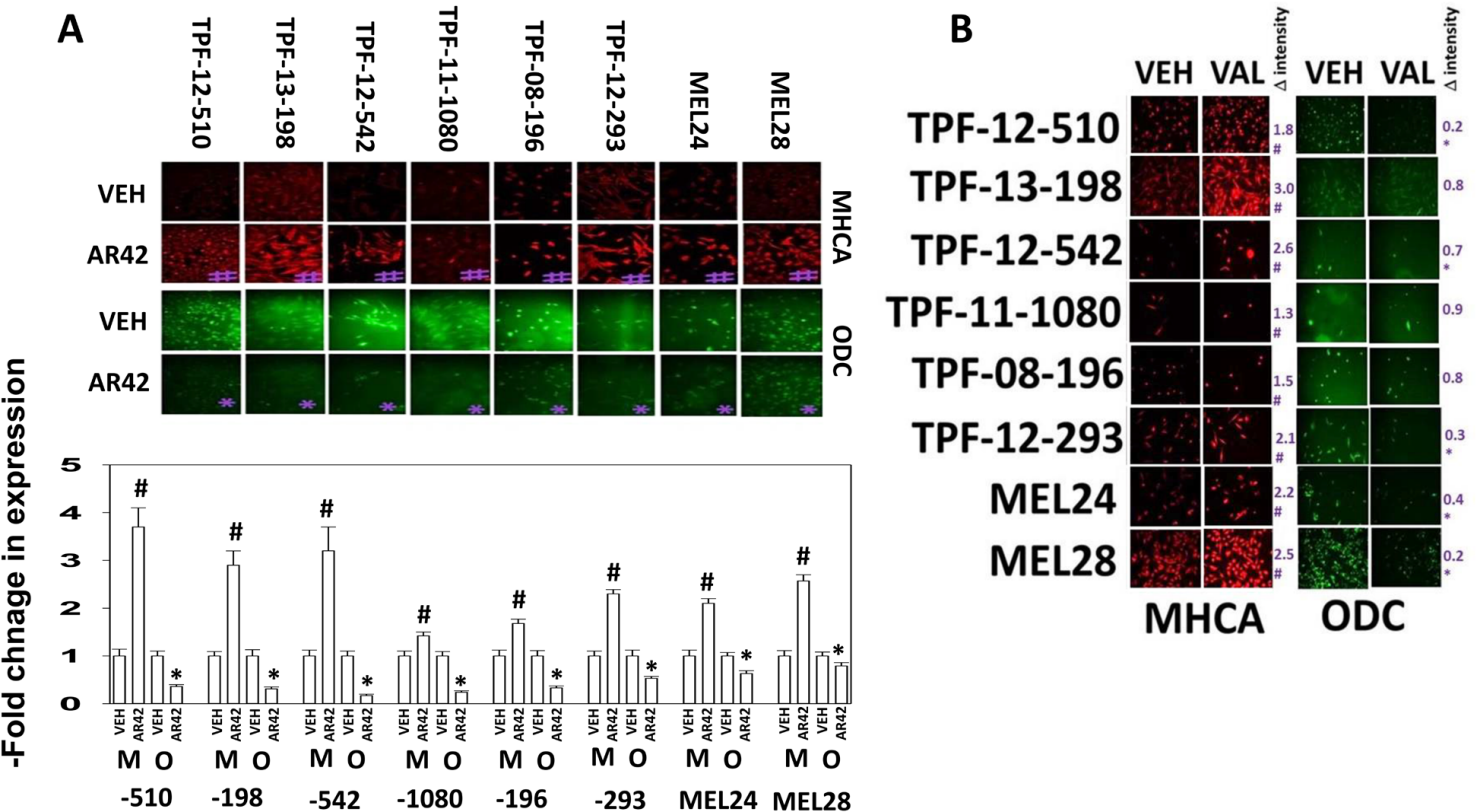
HDAC inhibitors regulate the expression of MHCA and ODC in melanoma cells **(A)** Melanoma cells were treated with vehicle control or with AR42 (600 nM) for 6h. Cells were fixed in place and immunostaining performed to determine the expression of MHCA and ODC (n = 3 +/-SEM). * p < 0.05 less than vehicle control; # p < 0.05 greater than vehicle control. **(B)** Melanoma cells were treated with vehicle control or with sodium valproate (250 μM) for 6h. Cells were fixed in place and immunostaining performed to determine the expression of MHCA and ODC (n = 3 +/-SEM). * p < 0.05 less than vehicle control; # p < 0.05 greater than vehicle control.

Melanoma was the first malignancy in which checkpoint inhibitory immunotherapy antibodies were approved, blocking the actions of PD1/PD-L1 and CTLA4. We discovered that AR42, at approximately 40% of its plasma C max within 6h reduced the expression of the immuno-regulatory proteins PD-L1 and to a lesser extent PD-L2 on PDX models of mutant B-RAF melanoma *in vitro*, which could in theory facilitate an immunological anti-tumor response (Figure 3A). Data showing a reduction in PD-L1 and PD-L2 expression in melanoma cells, using sodium valproate, were also observed (Figure 3B). AR42 and sodium valproate both increased the expression of the class I MHC molecule MHCA, which again could facilitate an innate immunological anti-tumor response (Figure 4A and 4B). AR42 and sodium valproate both reduced the expression of ornithine decarboxylase (ODC), which could de-repress an immunological anti-tumor response (Figure 4A and 4B). Similar data examining immuno-regulatory markers with both AR42 and sodium valproate was also obtained in multiple other tumor cell types (Supplementary Figures 2-5). Thus, AR42 not only efficaciously kills melanoma cells after 24h of exposure but it also likely predisposes them, within 6h of exposure, to being opsonized for killing using checkpoint inhibitor immunotherapy antibodies.

In studies examining the killing potential of AR42 over a 24h time course, we noted in the B16 mouse melanoma cell line that AR42 treated cells which were beginning to undergo cell death were shedding small vesicles reminiscent of those previously described in glioma cells [22]. We repeated these studies in human melanoma isolates 12h after AR42 exposure, a time point prior to the induction of single agent AR42 lethality (Figure 6; Supplementary Figures 7 and 8). Immuno-fluorescent staining of the vesicles from AR42 treated cells revealed that they contained the immunogenic protein HMGB1 and HSP70 (Figure 6A). In contrast, HSP90 did not exhibit a strong co-localization with HMGB1. Similar findings regarding HMGB1 extracellular release were made with AR42 and sodium valproate in multiple other tumor types (Supplementary Figures 7 and 8). Of note, HDAC inhibitors caused HMGB1 release from the PDX ovarian cancer isolate “Spiky” (Supplementary Figures 7 and 8) [23]. The patient from which the Spiky ovarian cancer cells were originally isolated was inherently platinum and taxane resistant, and died 4.5 months after her initial presentation.

**Figure 5:**
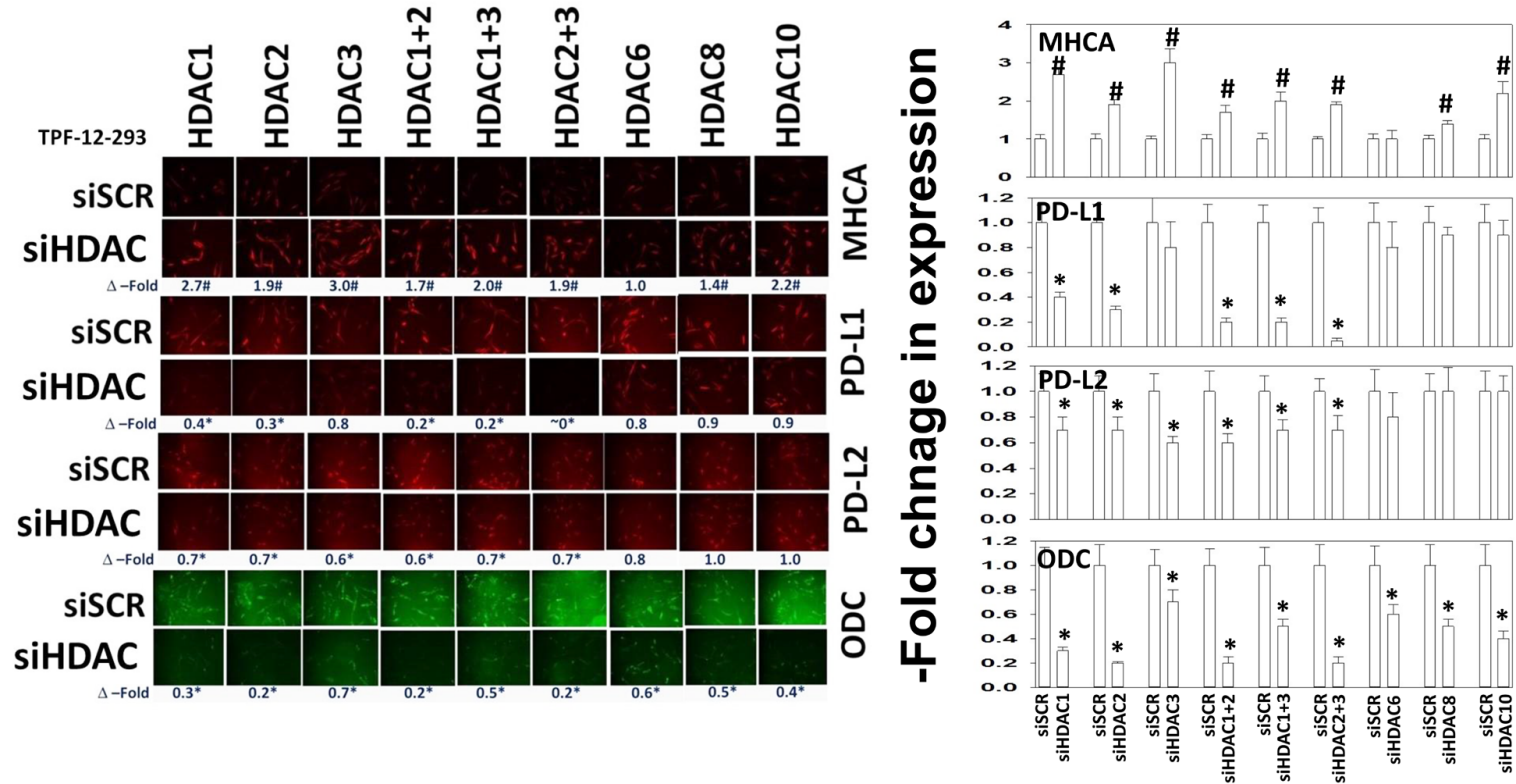
Molecular knock down of HDAC expression alters the expression of immune-modulatory markers TPF-12-293 cells were transfected with a scrambled siRNA (siSCR) or siRNA molecules to knock down: HDAC1, HDAC2, HDAC3, HDAC6, HDAC8 or HDAC10, alone or in the indicated combinations. Twenty-four h after transfection, cells were fixed in place and immunostaining performed to determine the expression levels of PD-L1, PD-L2, MHCA and ODC. (n = 3 +/-SEM). * p < 0.05 less than siSCR control; # p < 0.05 greater than siSCR control.

**Figure 6:**
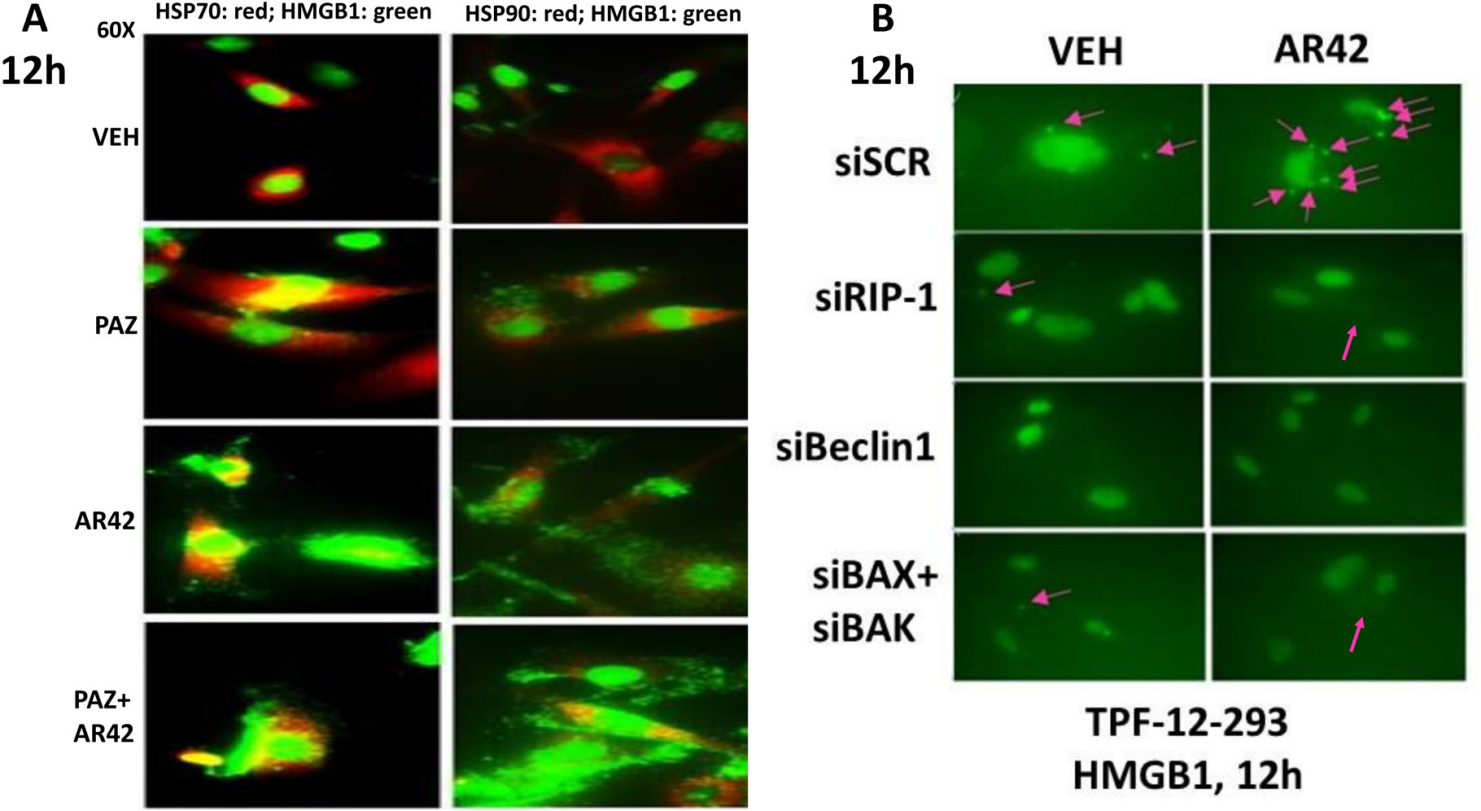
AR42 promotes the extracellular release of HMGB1 and HSP70 from melanoma cells **(A)** TPF-12-293 cells were treated with vehicle control or AR42 for 12h. Cells were fixed in place and immunostaining performed to detect the expression of HMGB1. The levels of extracellular HMGB1 vesicle staining were graded as: + (< 10% of cells); ++ (< 20% of cells); +++ (< 40% of cells); ++++ (> 60% of cells). **(B)** TPF-12-293 cells were transfected with a scrambled siRN A control or with siRNA molecules to knock down the expression of: RIP-1; Beclin1; and [BAX + BAK] combined. Twenty-four h after transfection cells were treated with vehicle control or with AR42 (600 nM) for 12h. Cells were fixed in place and immunostaining performed to determine the gross (60X mag.) expression and localization of HMGB1. Pink arrows indicate the observable extracellular vesicles.

Recent studies have suggested that death receptor signaling, *via* RIP-1 and RIP-3, could potentially regulate autophagosome formation and HMGB1 release [24]. Knock down of RIP-1 or of the toxic BH3 domain proteins [BAX+BAK] did not alter basal levels of vesicle shedding but did reduce the stimulation of vesicle production caused by [pazopanib + AR42] (Figure 6B, p < 0.05). Knock down of Beclin1, however, abolished both basal levels of HMGB1 positive vesicle formation as well as drug-stimulated HMGB1 vesicle production. Thus, both AR42 and sodium valproate also likely have the capacity to immunologically compromise mutant B-RAF melanoma cells and other tumor cell types *via* HMGB1 release. Additional studies beyond the scope of the present manuscript will be required to fully characterize the vesicles being released from the drug-treated melanoma cells [22–25].

Many studies examining the therapeutic actions of HDAC inhibitors do not in parallel determine the key HDAC protein effectors in the biological actions of their drugs. Hence, we determined which HDACs or combinations of HDACs regulated expression of PD-L1, PD-L2, MHCA and ODC in melanoma cells and ovarian cancer cells. Knock down of HDAC1 and HDAC3 enhanced the expression of MHCA (Figure 5 and Supplementary Figure 9). Knock down of HDAC1 and HDAC2 reduced the expression of PD-L1; knock down of HDAC1-3, HDAC8 and HDAC10 reduced the expression of PD-L2; knock down of HDAC1 and HDAC2, as well as knock down of HDAC2 and HDAC3 reduced the expression of ODC. However, the data in the two cell lines tested also reveals some inconsistencies for several of the immunogenic markers that could be the result of experimental (biological) variance, or complex compensatory mechanisms involving several HDACs. Collectively, the data presented in this figure argues that MHCA, PD-L1/L2 and ODC are regulated by multiple/overlapping HDACs. This suggests that pan-HDAC inhibition may be more effective than specific HDAC inhibition.

**Figure 7:**
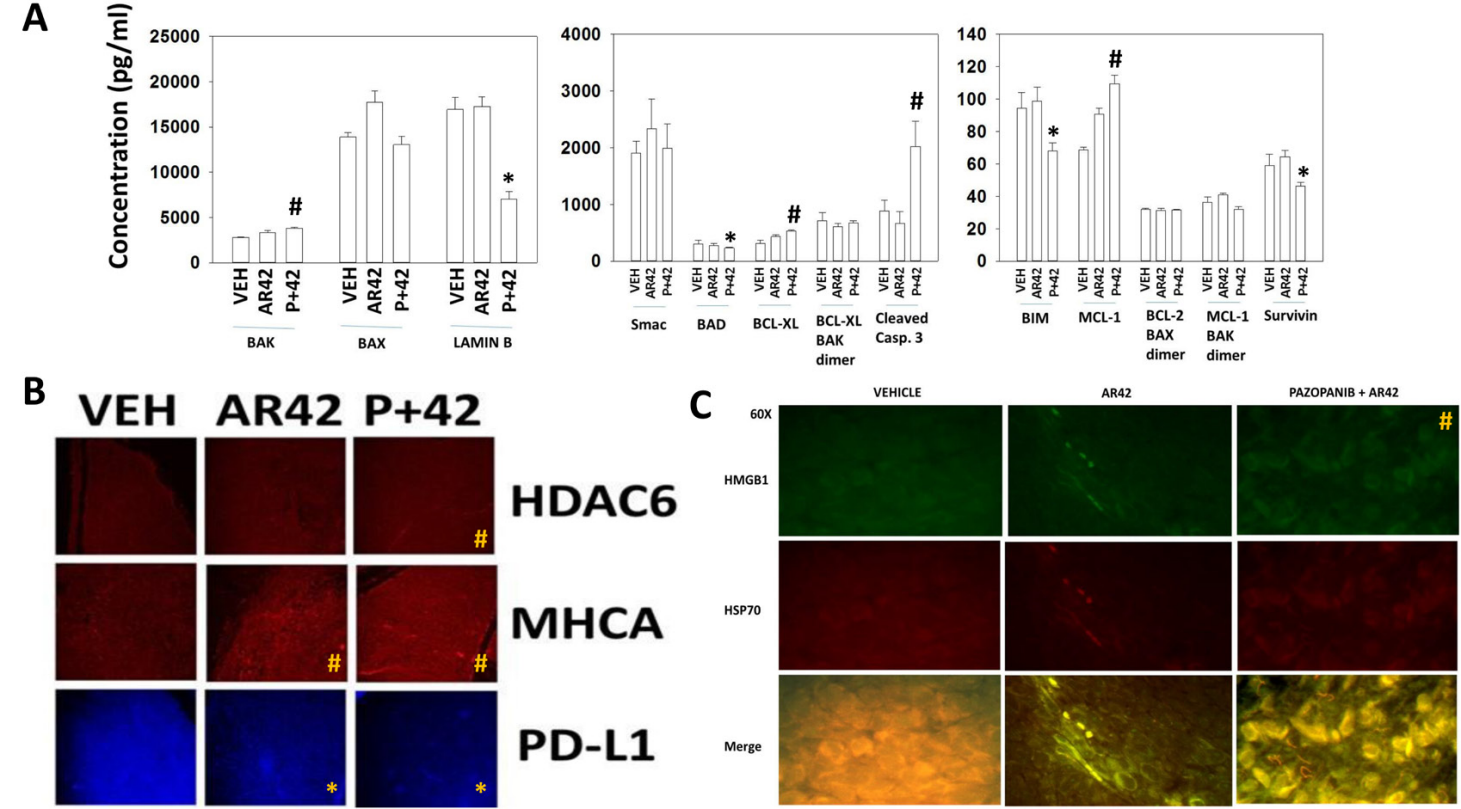
Tumors previously exposed to [pazopanib + AR42] exhibit higher basal caspase 3 activity that is associated with increased MHCA expression and greater (HMGB1-HSP70) extracellular release **(A)** MEL28-R tumors were isolated at the time of animal nadir (∼250 mm^3^). Tumor lysates were prepared per the manufacturer’s instructions and multiplex assays performed on a Bio-Rad MAGPIX machine to determine the expression of the indicated proteins (n = 3 separate tumors +/-SEM) * p < 0.05 less than vehicle treated tumor value; # p < 0.05 greater than vehicle treated tumor value. **(B)** MEL28-R tumors isolated at the time of animal nadir were fixed, embedded in paraffin and 4 μm sections obtained. Sections were de-paraffinized, renatured and stained to examine the gross (10X mag.) expression of HDAC6, MHCA and PD-L1. (n = 3 separate tumors +/-SEM) * p < 0.05 less than vehicle treated tumor value; # p < 0.05 greater than vehicle treated tumor value. **(C)** MEL28-R tumors isolated at the time of animal nadir were fixed, embedded in paraffin and 4 μm sections obtained. Sections were de-paraffinized, renatured and stained to examine the gross (60X mag.) expression and co-localization of HMGB1 and HSP70. (n = 3 separate tumors +/-SEM) # p < 0.05 greater than vehicle treated tumor value.

In our prior melanoma studies using trametinib/dabrafenib resistant MEL28 tumors we discovered that pazopanib and AR42 interacted to prolong animal survival [1]. Using multiplex assays on tumor material isolated at the time of humane sacrifice, several weeks after drug exposure, we assessed the relative levels and activities of protective and toxic BH3 domain proteins. Tumors previously exposed to [pazopanib + AR42] had increased their expression of BAK, BCL-XL, MCL-1 and of the cleaved active form of caspase 3 (Figure 7A). The expression of Lamin B (a caspase 3 substrate), BAD and survivin was reduced in tumors previously exposed to [pazopanib + AR42]. In parallel studies, we performed immunohistochemistry on dabrafenib/trametinib resistant MEL28 tumors previously exposed to AR42 or [pazopanib + AR42]. The expression of HDAC6, previously shown to be rapidly down-regulated *in vitro* by AR42, was modestly enhanced 40 days after drug exposure (Figure 7B). The expression level of the class I MHC protein MHCA was elevated, and the expression of the immuno-inhibitory protein PD-L1 was reduced. Prior treatment of tumors with AR42 reduced the expression of HSP70 and HMGB1 (Figure 7C). Prior exposure of tumors to [pazopanib + AR42], however, resulted in elevated HSP70 and HMGB1 expression and co-localization, with the extracellular presence of vermiform aggregates of immunogenic HSP70.

**Figure 8:**
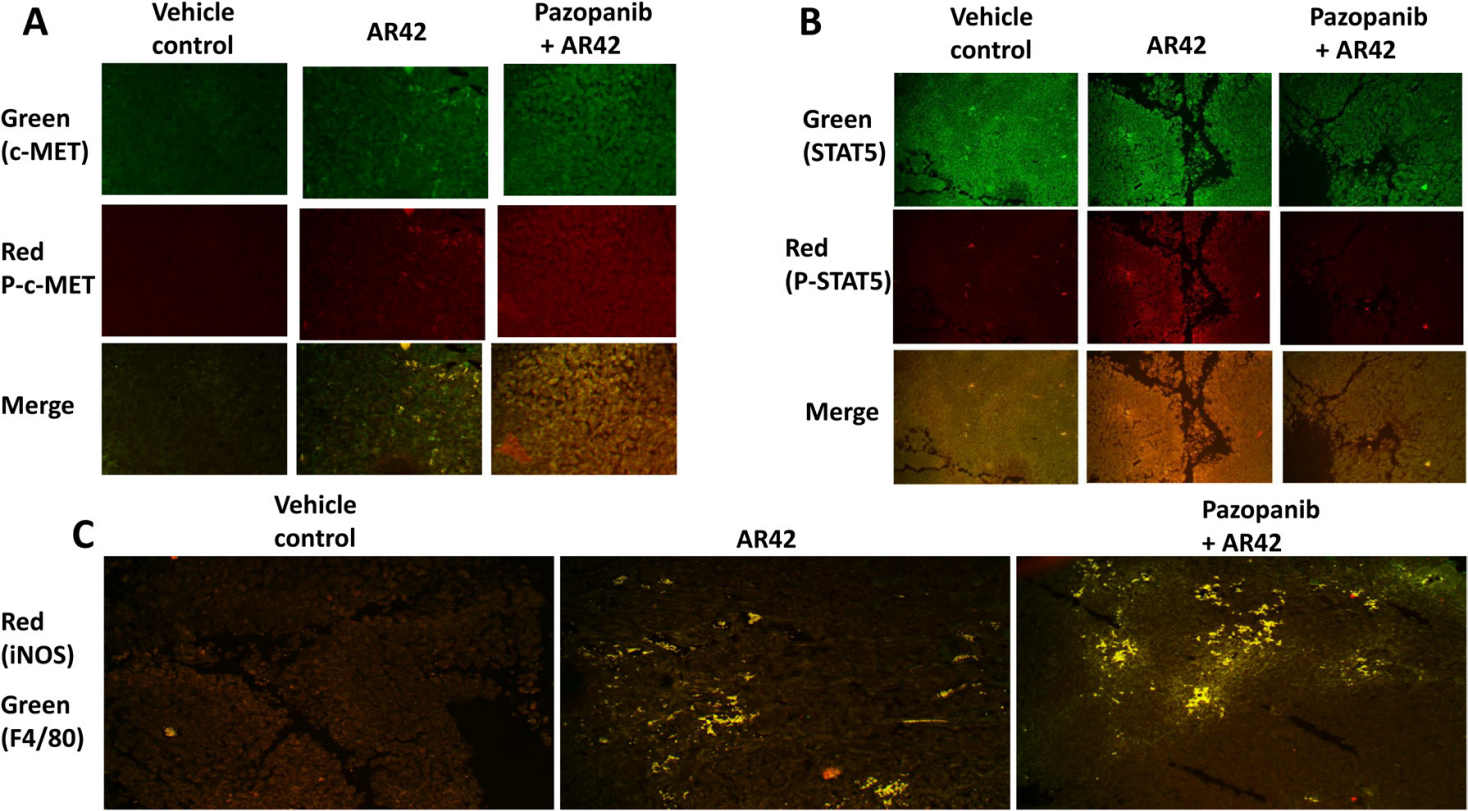
Drug-resistant melanoma cells up-regulate c-MET after [pazopanib + AR42] exposure **(A** & **B)** MEL28-R tumors, isolated at the time of animal nadir, were fixed, embedded in paraffin and 4 μm sections obtained. Sections were de-paraffinized, renatured and stained to examine the gross (10X mag.) expression and phosphorylation of c-MET and STAT5. (n = 3 separate tumors +/-SEM). **(C)** MEL28-R tumors isolated at the time of animal nadir were fixed, embedded in paraffin and 4 μm sections obtained. Sections were de-paraffinized, renatured and stained to examine (10X mag.) the colocalization of F4/80 staining and iNOS staining.

In Booth *et al.* we had demonstrated that dabrafenib/trametinib resistant MEL28 cells, after *in vivo* exposure to [pazopanib + AR42], no-longer relied on ERBB receptor signaling for survival and had switched their survival signaling to be mediated through the HGF receptor, c-MET [1]. Compared to vehicle control treated tumors, MEL28-R tumors exposed to [pazopanib + AR42] had evolved to over-express c-MET which was associated with increased c-MET protein phosphorylation (Figure 8A). After exposure to [pazopanib + AR42], the total expression of STAT5 declined in the tumor cells. Nevertheless, in tumors exposed to [pazopanib + AR42] the percentage of STAT5 being phosphorylated was enhanced (p < 0.05). Drug exposed tumors also exhibited elevated staining for M1 phenotype F4/80+ iNOS+ macrophages, that in theory would act in an anti-tumor fashion to suppress growth (Figure 8C). Our prior studies had shown STAT3 activation was an evolutionary survival response in [pazopanib + AR42] treated melanoma tumors; this, together with our present findings, argues that a c-MET – STAT3/STAT5 signaling pathway may be a key evolutionary survival signaling module that is selected for in melanoma after [pazopanib + AR42] exposure [1].

**Figure 9:**
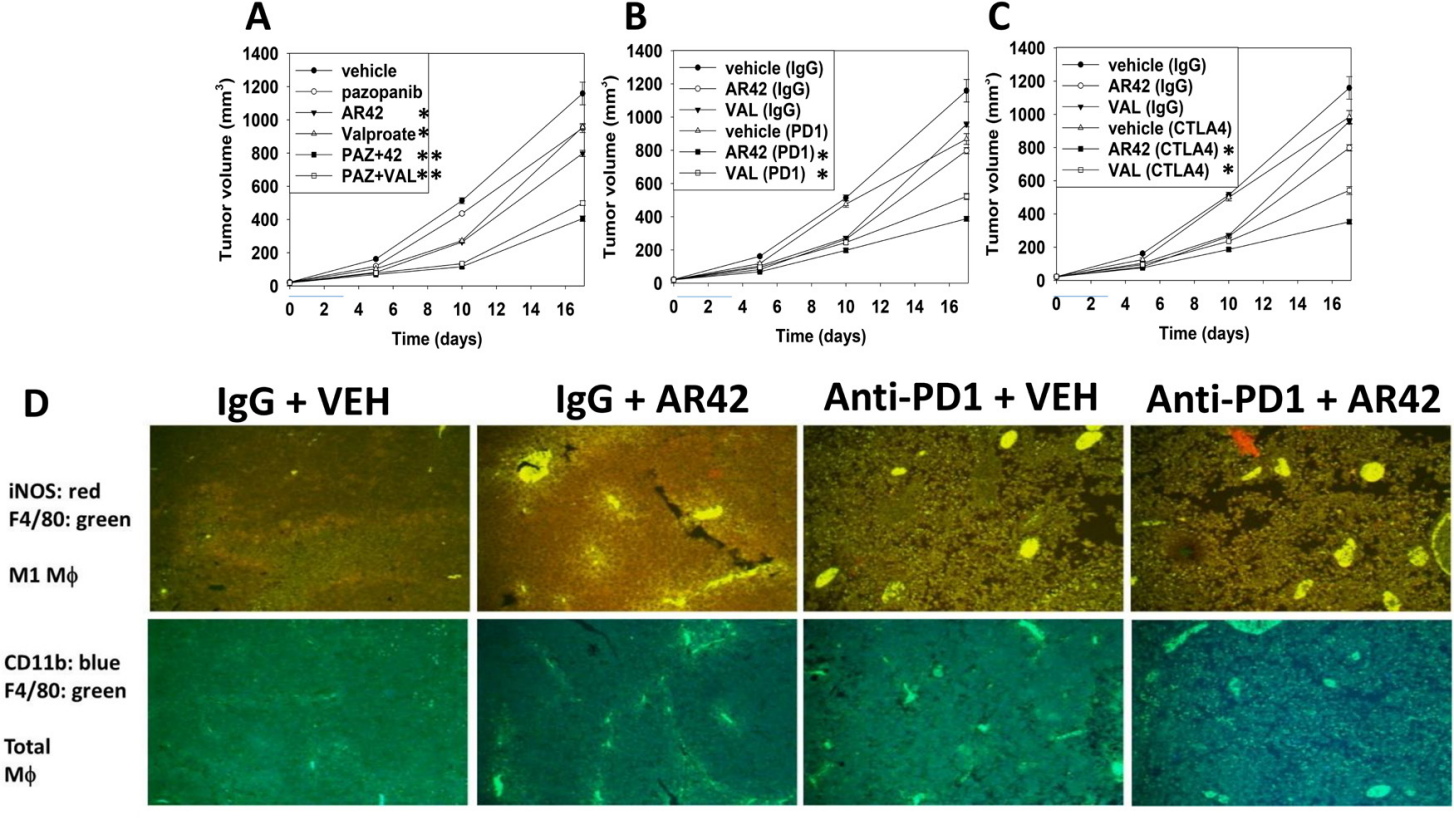
[Pazopanib + AR42/valproate] interact to reduce tumor growth; both valproate and AR42 enhance the anti-tumor effects of an anti-PD-1 and of an anti-CTLA4 antibody **(A-C)** B16 mouse melanoma cells (1 × 10^6^) were implanted into the rear flanks of C57 black mice and tumors permitted to form for 6 days until the mean tumor volume was ∼25 mm^3^. Animals were then segregated into groups with near identical mean volumes and the animals then treated for three days with the indicated therapeutic agents: vehicle control (cremophore); AR42 15 mg/kg (Days 1 and 3); pazopanib 25 mg/kg (Days 1, 2, 3); sodium valproate 50 mg/kg (Days 1, 2, 3) or in combination. Two days after cessation of drug exposure animals are injected IP with: a control IgG (100 μg); an anti-PD-1 IgG (100 μg); or an anti-CTLA4 IgG (100 μg). Tumor volumes were measured prior to drug administration and after the initiation of therapeutic interventions. (n = 10 mice per group +/-SEM). **(D)** As the tumor volume of an animal reached 1,500 mm^3^, animals were humanely sacrificed and the plasma and tumor from each animal collected. Tumors were fixed, embedded in paraffin, 5 μm sections taken, the section de-paraffinized and re-natured. Immunostaining was performed with the indicated antibodies to detect total macrophage levels and M1 macrophage levels in the tumor.

We next performed animal studies to validate or refute our *in vitro* findings. One million B16 mouse melanoma tumor cells were implanted into the rear flanks of C57 black mice and five days later when tumor volumes were 23.2 +/- 0.8 mm^3^animals were treated with AR42/valproate and/or the multi-kinase inhibitor pazopanib for three days. Animals, 48h after AR42/valproate exposure, were then treated with a control non-specific antibody, an anti-PD-1 antibody or an anti-CTLA-4 antibody. Vehicle control treated tumors rapidly grew and within 17 days of implantation tumor volumes had begun to reach the sacrifice cut-off point of 1,500 mm^3^. A 1,500 mm^3^ tumor in a 20-gram mouse is the equivalent of a 4.5 kg tumor in a 60-kg human (Figures 9A-9C). Humane animal sacrifice in the mono-therapy groups was predicated on the central portions of their tumors rapidly becoming necrotic and ulcerated. Tumor growth was significantly reduced in tumors exposed to either [pazopanib + AR42] or [pazopanib + valproate] (Figure 9A). AR42 and sodium valproate both significantly enhanced the anti-tumor efficacy of an anti-PD-1 antibody and of an anti-CTLA4 antibody (Figure 9B and 9C). At the time of humane animal sacrifice sections of tumor material were made for immuno-histochemical analyses, and other portions of tumor material lysed to enable multiplex analyses to performed. B16 tumors exposed to [pazopanib + HDAC inhibitor] exhibited greater infiltration of F4/80+ macrophages that expressed iNOS, indicative of the M1 macrophage phenotype (Figure 9D).

**Figure 10:**
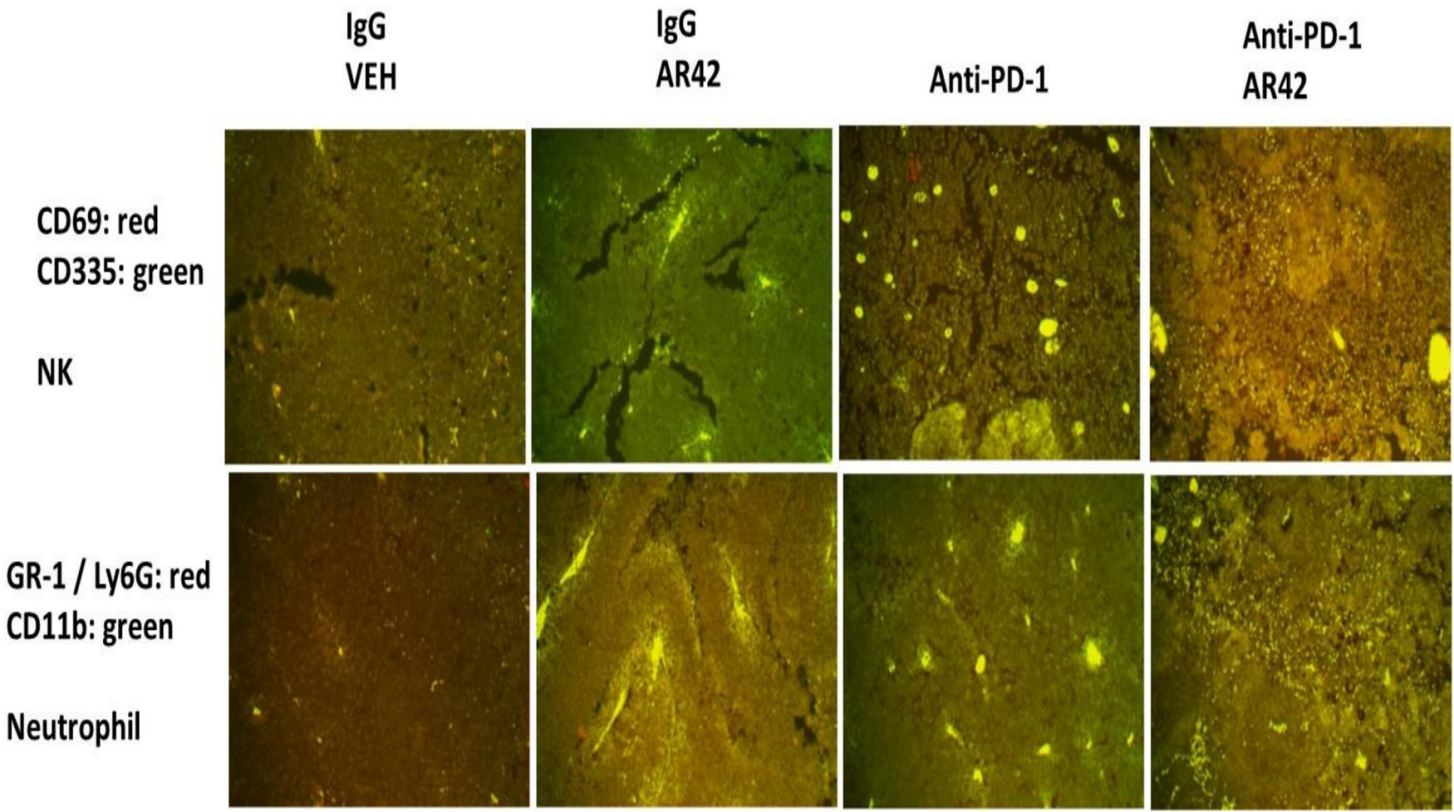
Natural Killer and neutrophil levels in drug treated tumors (1) As the tumor volume of an animal reached 1,500 mm^3^, animals were humanely sacrificed and the plasma and tumor from each animal collected. Tumors were fixed, embedded in paraffin, 5 μm sections taken, the section de-paraffinized and re-natured. Immunostaining was performed with the indicated antibodies: **Upper.** CD69 and CD335 to detect Natural Killer cells; **Lower.** GR1 + CD11b to detect neutrophils.

**Figure 11:**
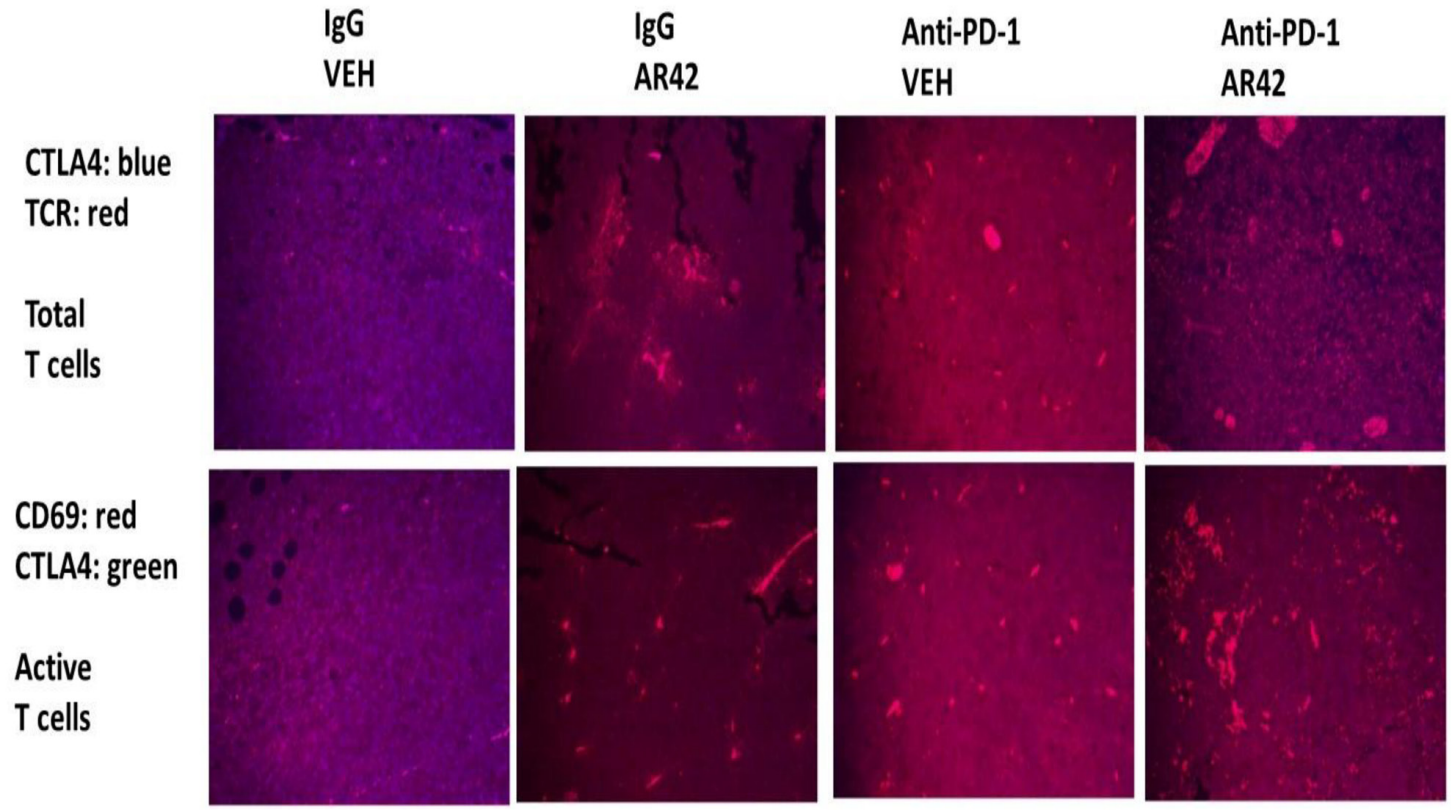
Total and activated T cell levels in drug treated tumors As the tumor volume of an animal reached 1,500 mm^3^, animals were humanely sacrificed and the plasma and tumor from each animal collected. Tumors were fixed, embedded in paraffin, 5 μm sections taken, the section de-paraffinized and re-natured. Immunostaining was performed with the indicated antibodies: **Upper.** CTLA4 and the T Cell Receptor to detect total T cell levels; **Lower.** CD69 and CTLA4 to detect activated T cells.

**Figure 12:**
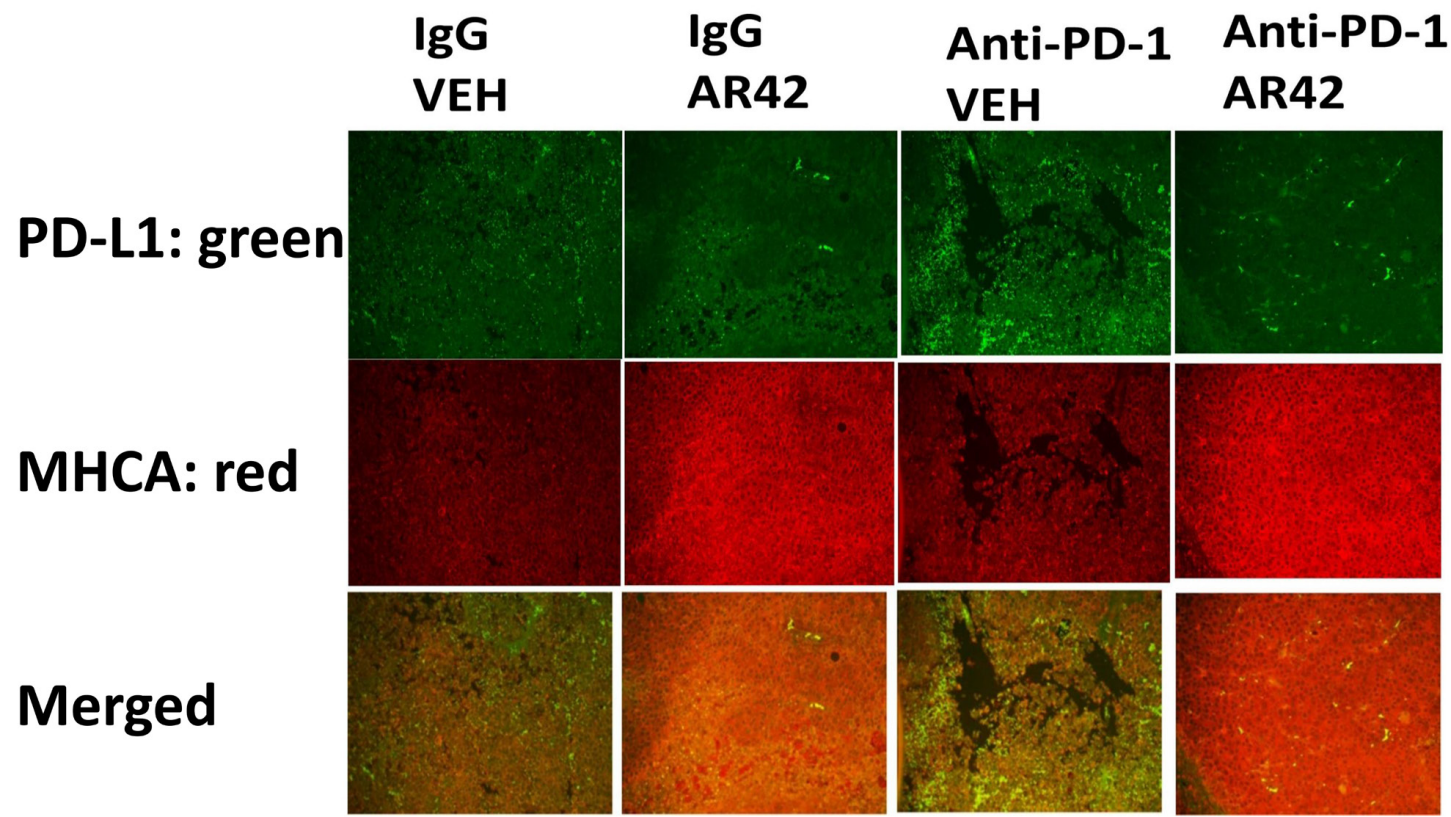
To detect MHCA and PD-L1 levels in drug treated tumors As the tumor volume of an animal reached 1,500 mm^3^, animals were humanely sacrificed and the plasma and tumor from each animal collected. Tumors were fixed, embedded in paraffin, 5 μm sections taken, the section de-paraffinized and re-natured. Immunostaining was performed with the indicated antibodies; **Upper.** Green staining to detect PD-L1 levels; **Lower.** Red staining for MHCA.

Exposure of B16 tumors to the HDAC inhibitor AR42 or to an anti-PD-1 antibody increased the tumor localized levels of NK cells, neutrophils and activated T cells. Of interest, in contrast to the individual agents, combined exposure of tumors to AR42 and the anti-PD-1 antibody resulted in the wide dispersion of the macrophages, NK cells, neutrophils and activated T cells within the tumor (Figures 10-12). Identical data examining immune cell markers in the drug-exposed tumors were obtained using the pan-HDAC inhibitor sodium valproate (Supplementary Figures 10-13).

**Figure 13:**
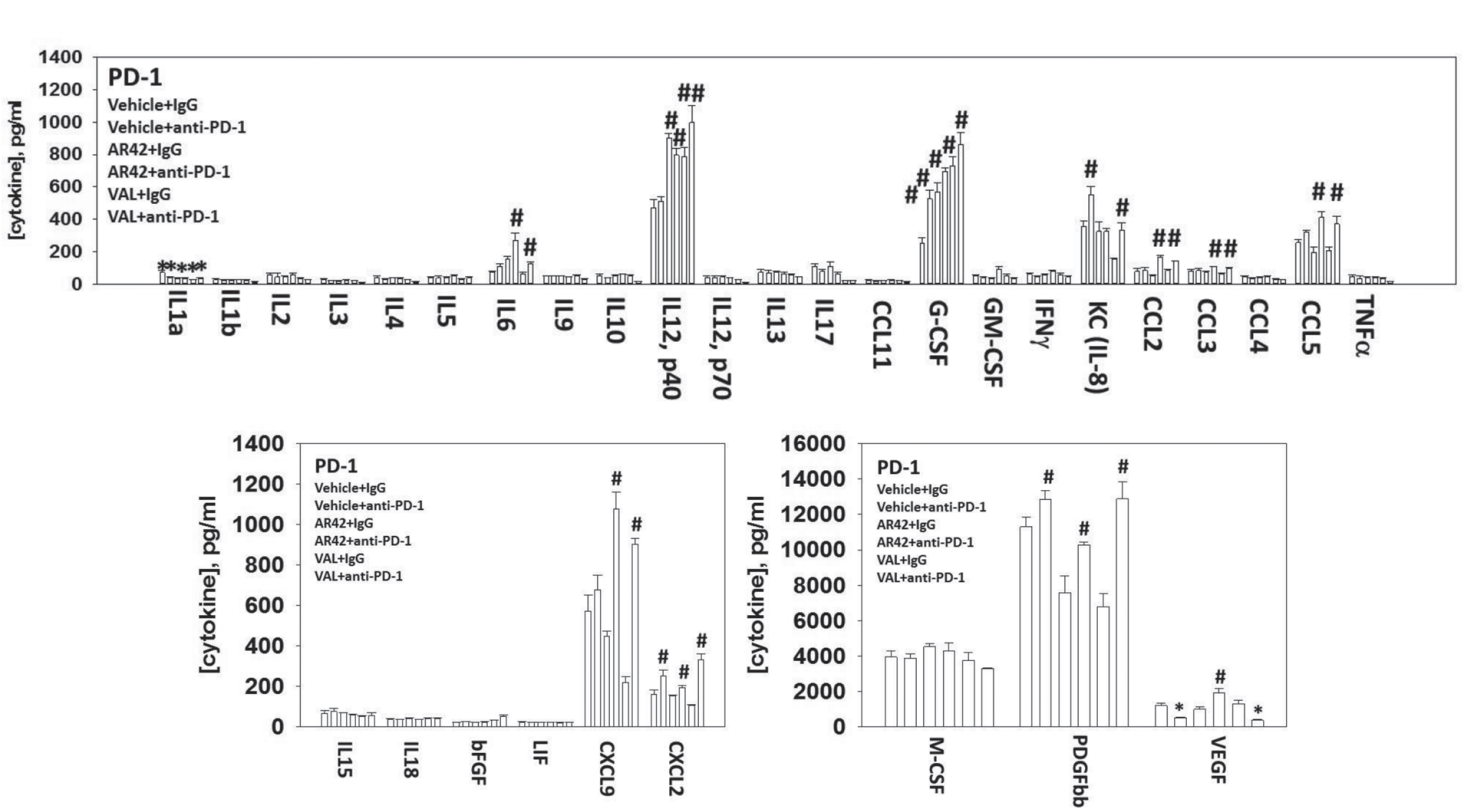
Multiplex data comparing vehicle control to HDAC inhibitor in the presence or absence of an anti-PD-1 antibody Plasma from animals treated with: vehicle control IgG; vehicle control anti-PD-1; AR42 IgG; AR42 anti-PD-1; sodium valproate IgG; sodium valproate anti-PD-1; was processed in the MAGPIX multiplex machine to determine the concentrations of the indicated cytokines (n = 3 separate tumors +/-SEM) * p < 0.05 less than vehicle control; # p < 0.05 greater than vehicle control.

Plasma from animals carrying B16 tumors, previously exposed to [HDAC inhibitor + anti-PD-1 antibody] displayed elevated IL-6, IL-12, CCL2, CCL3, CCL5, CXCL9 and CXCL2 levels and reduced IL1a levels (Figure 13). The changes in cytokine expression using an anti-CTLA4 antibody appeared to be HDAC inhibitor dependent with a trend for the expression of IL6 and CCL5 expression being elevated and that of G-CSF and CXCL9 being reduced (Supplementary Figures 14 and 15). In plasma from animals treated with checkpoint inhibitors and AR42 the plasma concentration of TGF beta was elevated, an effect not observed using sodium valproate (Supplementary Figure 16). The increase in IL-6, IL-12, CCL2, CCL3, CCL5, CXCL9 levels would *a priori* predict for elevated levels of M1 type macrophages in the tumor, which independently validates our prior IHC staining findings.

**Figure 14:**
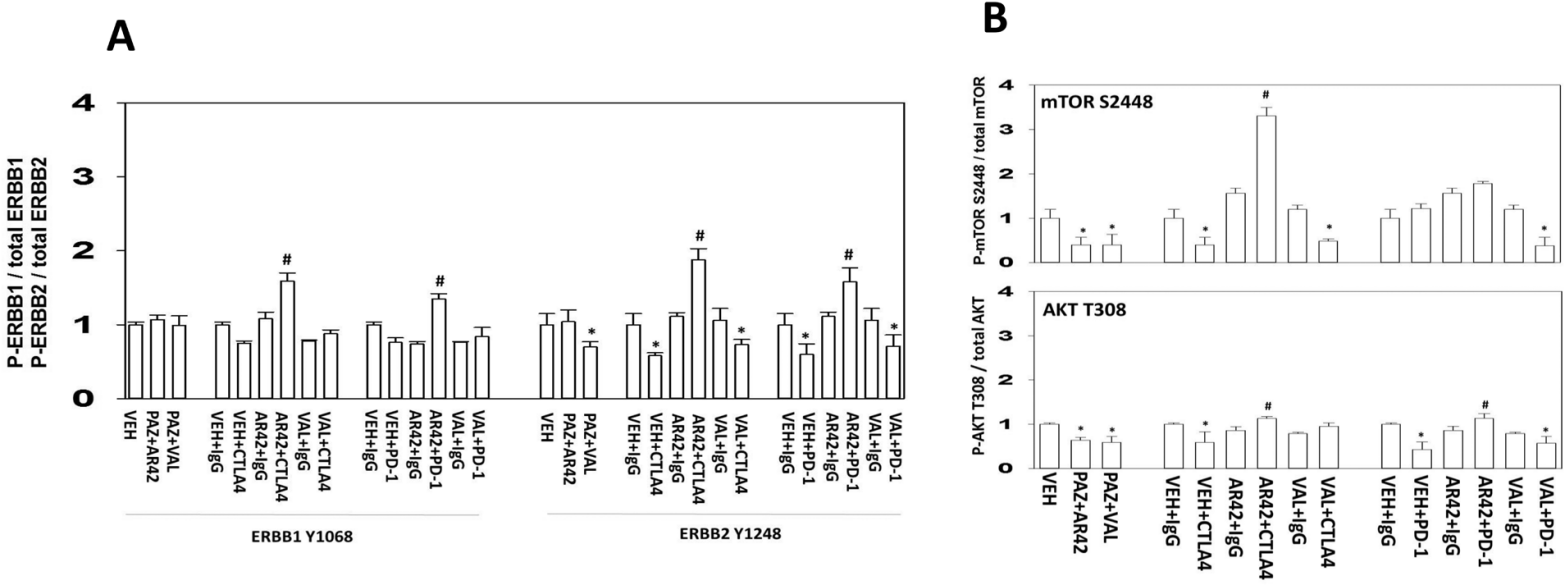
AR42 plus immunotherapy enhances the phosphorylation of ERBB1 and ERBB2 in surviving tumor cells **(A)** Tumor material from animals treated with: vehicle control IgG; vehicle control anti-PD-1; AR42 IgG; AR42 anti-PD-1; sodium valproate IgG; sodium valproate anti-PD-1; was lysed and processed in the MAGPIX multiplex machine to determine the phosphorylation of the indicated proteins, ERBB1 and ERBB2 (n = 3 separate tumors +/-SEM) * p < 0.05 less than vehicle control; # p < 0.05 greater than vehicle control. **(B)** Tumor material from animals treated with: vehicle control IgG; vehicle control anti-PD-1; AR42 IgG; AR42 anti-PD-1; sodium valproate IgG; sodium valproate anti-PD-1; was lysed and processed in the MAGPIX multiplex machine to determine the phosphorylation of the indicated proteins, mTOR S2448 (mTORC1) and AKT T308 (n = 3 separate tumors +/-SEM) * p < 0.05 less than vehicle control; # p < 0.05 greater than vehicle control.

We then examined the phosphorylation/activities of intracellular tumor signal transduction proteins. The activities of many signaling proteins, previously analyzed in the MEL28-R model and shown to be altered, were unchanged in the B16 tumors [1]. Unexpectedly, the combination of AR42, but not sodium valproate, with checkpoint immunotherapies activated both ERBB1 and ERBB2 in the surviving tumor cells (Figure 14A). These observations also correlated with increased mTOR activity and to a lesser extent with increased AKT activity, and with reduced PTEN expression (Figure 14B; Supplementary Figure 17). The role of altered PTEN expression levels in the biology of B16 melanoma cells will require studies beyond the scope of the present manuscript.

## DISCUSSION

The present studies were initially proposed to determine whether the drug combination of pazopanib and AR42/sodium valproate, previously shown to be an efficacious way to kill sarcoma, renal cancer and melanoma cells and now an open phase I trial, could also opsonize melanoma cells to immunotherapy checkpoint inhibitory antibodies.

HDAC inhibitors as single agents, *via* modulation of HDAC1, HDAC3, HDAC8 and HDAC10 function, increased the expression of MHCA and decreased the expression of PD-L1, PD-L2 and ODC on tumor cells. Transient exposure of tumors to AR42 or sodium valproate enhanced the anti-tumor efficacy of a subsequent anti-PD-1 antibody and of an anti-CTLA4 antibody. There is at present an open clinical trial combining the HDAC6 specific HDAC inhibitor ACY-241 with Ipilimumab (anti-CTLA4) and Nivolumab (anti-PD-1) for melanoma (NCT02935790). It is unclear whether in this trial the HDAC inhibitor is dosed prior to the immunotherapy or whether HDAC inhibitor dosing is continuous. This is of importance as it is well known in the clinic that prolonged exposure to HDAC inhibitors can lead to reduced hematopoiesis with thrombocytopenia and general myelosuppression. This would be predicted to lower the anti-tumor response of any immunotherapy. In addition, our data, knocking down individual HDAC proteins, argues that a highly specific HDAC6 inhibitor in melanoma cells is unlikely to effectively modulate the expression of PD-L1, PD-L2, MHCA or ODC in a manner like AR42 or valproate. Collectively, our findings strongly argue that transient exposure to pan-HDACIs, such as AR42 and sodium valproate, will opsonize melanoma cells to immunotherapy checkpoint inhibitor approaches.

In a recent manuscript using dabrafenib/trametinib resistant human MEL28 cells growing in athymic mice we demonstrated that pazopanib and AR42 interacted to profoundly suppress melanoma tumor growth which resulted in prolonged animal survival [1]. Our present findings, using mouse B16 melanoma cells in their syngeneic immune competent C57 black mouse host, confirm and extend our prior data to demonstrate that pazopanib and sodium valproate also interact *in vivo* to suppress melanoma tumor growth. Previously we had presented similar *in vivo* finings combining pazopanib and sodium valproate using HT1080 human sarcoma cells grown in athymic mice [6].

The molecular mechanisms by which HDAC inhibitors act to modulate protein expression and tumor cell survival/cell death are complex. We previously published that [pazopanib + HDAC inhibitor] treatment reduced the expression level of HDAC6 in a wide variety of tumor cell types. In melanoma cells knock down of [HDAC6 + HDAC2] or [HDAC6 + HDAC10] significantly enhanced pazopanib lethality whereas knock down of [HDAC6 + HDAC1] or [HDAC6 + HDAC3] were less effective [1]. HDACs1-3 have been argued to suppress the PD-L1 and PD-L2 promoters [26]. HDAC1 has been proposed to suppress the Class I MHC promoter, MHCA, as well as associate with the ODC promoter [27–30]. HDAC6 has been shown to be essential for melanoma cell cycle progression [27]. The impact of lower ODC expression may influence the activity of macrophages in the tumor; lower ODC levels in tumor cells facilitate anti-tumor M1 macrophage activation [31]. In the present studies we discovered that knock down of HDAC1 +/- HDAC2 +/- HDAC3 regulated the expression of PD-L1, PD-L2, MHCA and ODC. HDAC6 knock down appeared to diminute the regulatory effects of altered HDAC1/2/3 expression on the levels of PD-L1, PD-L2, MHCA and ODC. Experiments beyond the scope of the present studies, linking HDAC6 and HSP90/HSP70 chaperone functionality, with the promoter regulatory biology of HDACs1/2/3, are beyond the scope of the present manuscript.

Compared to many other tumor types previously exposed to HDAC inhibitors in the Dent laboratory, e.g. breast cancer; liver cancer; pancreatic carcinoma; sarcoma; renal carcinoma; glioblastoma; the PDX and established mutant B-RAF melanoma isolates tested in our studies were highly sensitive to being killed by HDAC inhibitors as single agents at clinically relevant concentrations. B16 mouse melanoma cells express a wild type B-RAF and were as sensitive to being killed as the PDX models suggesting that the sensitivity is inherent in the biology of melanoma tumor cells. Our *in vitro* data was confirmed in our animal studies where both AR42 and valproate significantly reduced tumor growth. We also presented evidence of rapid efficacious killing of melanoma cells by the FDA approved HDAC inhibitors vorinostat, entinostat, panobinostat, as well as by sodium valproate. Our aim, in the very near future, is to propose a new phase I trial in refractory melanoma patients with [pazopanib + valproate] and checkpoint inhibitor antibodies.

Multiplex analyses on plasma isolated from vehicle and [pazopanib + HDAC inhibitor] treated mice demonstrated that tumors previously exposed to [pazopanib + HDAC inhibitor] had elevated IL5 plasma levels and reduced plasma levels of IL10, IL13, IL17, GM-CSF, interferon γ, CCL4, CXCL9 and PDGF. IL5, IL13, IL10, GM-CSF and PDGF are tumorigenic cytokines and GM-CSF, IL5, IL10 and IL13 are known to regulate T-helper cells [32, 33]. Elevated IL5 levels would be predicted to enhance type 2 T-helper cell activity whilst reduced IL10 levels would facilitate type 1 T-helper cell activity. Plasma from animals exposed to [HDAC inhibitor + anti-PD-1] had elevated levels of IL-6, IL-12, CCL2, CCL3, CCL5, CXCL9 and CXCL2. CXCL9 and CCL5 can directly promote tumor growth but the cytokines are also a chemo-attractant which promotes T cell and monocyte infiltration into the tumor microenvironment and the development of T memory cells [34–37]. In melanoma, elevated tumor levels of T lymphocytes are associated with a better prognosis and elevated expression of CXCL9 and CCL5 has been associated with a better response in metastatic melanoma patients. Similar data, including CCL2, has also been reported as correlating with a better prognosis for melanoma patients.

Some of the altered biology in the previously drug exposed tumors were unexpected. Notably, that AR42 combined with immunotherapy to activate ERBB1, ERBB2, AKT and mTOR. In contrast, sodium valproate did not cause the tumor cells to evolve in this manner. The expression level of the tumor-suppressor PTEN weakly correlated with the observed changes in mTOR and AKT phosphorylation, and effect that also could not be explained by changes in the phosphorylation of the regulatory site PTEN S380. Valproate, but not AR42, as a single agent reduced the levels of TGF beta which would further facilitate the anti-tumor actions of immunotherapy. The reasons for the differential effects of AR42 and sodium valproate are presently unclear, but possible mechanisms include altered autophagy flux and the more potent HDAC inhibitory activity of AR42.

In conclusion, we have proven that melanoma cells *in vivo* are sensitive to the anti-tumor effects of HDAC inhibitors, an effect that can be enhanced by the multi-kinase and chaperone inhibitor pazopanib. Tumors that regrew after exposure contained elevated levels of anti-tumor M1 macrophages. Transient exposure of tumors to pan-HDAC inhibitors opsonized melanoma tumors to the anti-tumor actions of anti-PD-1 and anti-CTLA4 antibodies. Tumors that regrew after exposure had elevated M1 macrophage levels as well as elevated levels of activated cytotoxic T cells. Our future goals are to translate our findings into trials for recurrent melanoma patients.

## MATERIALS AND METHODS

### Materials

Pazopanib, AR42 and all other HDAC inhibitors were purchased from Selleckchem (Houston, TX). Sodium valproate was from Sigma (St. Louis, MO). Trypsin-EDTA, DMEM, RPMI, penicillin-streptomycin were purchased from GIBCOBRL (GIBCOBRL Life Technologies, Grand Island, NY). MEL24, MEL28, A549, H460 and H1975 cells were purchased from the ATCC and were not further validated beyond that claimed by ATCC. Cells were re-purchased every ∼6 months. Characterized PDX melanoma isolates were from the University of Pittsburgh’s melanoma cell bank. H&N PDX tumor isolates were kindly provided by Dr. John Lee (Chan Soon-Shiong Institute of Molecular Medicine, Culver City, CA). ADOR cells were a personal gift to the Dent lab from a female NSCLC patient [7]. Commercially available validated short hairpin RNA molecules to knock down RNA/protein levels were from Qiagen (Valencia, CA) (Supplementary Figure 18) [1]. Control IgG, anti-PD-1 and anti-CTLA4 endotoxin-free antibodies were purchased from Bio-Xcell (West Lebanon, NH). Reagents and performance of experimental procedures were described in refs: [1–8].

### Methods

#### Culture and *in vitro* exposure of cells to drugs

All cell lines were cultured at 37 °C (5% (v/v CO_2_) *in vitro* using RPMI supplemented with dialyzed 5% (v/v) fetal calf serum and 10% (v/v) Non-essential amino acids. For short term cell killing assays, immunoblotting studies, cells were plated at a density of 3 × 10^3^ per cm^2^ (∼2 × 10^5^ cells per well of a 12 well plate) and 48h after plating treated with various drugs, as indicated. *In vitro* drug treatments were generally from a 100 mM stock solution of each drug and the maximal concentration of Vehicle carrier (VEH; DMSO) in media was 0.02% (v/v). Cells were not cultured in reduced serum media during any study in this manuscript.

### Transfection of cells with siRNA or with plasmids

#### For plasmids

Cells were plated and 24h after plating, transfected. Plasmids expressing a specific mRNA (or siRNA) or appropriate vector control plasmid DNA was diluted in 50μl serum-free and antibiotic-free medium (1 portion for each sample). Concurrently, 2μl Lipofectamine 2000 (Invitrogen), was diluted into 50μl of serum-free and antibiotic-free medium (1 portion for each sample). Diluted DNA was added to the diluted Lipofectamine 2000 for each sample and incubated at room temperature for 30 min. This mixture was added to each well/dish of cells containing 200μl serum-free and antibiotic-free medium for a total volume of 300 μl, and the cells were incubated for 4 h at 37 °C. An equal volume of 2x medium was then added to each well. Cells were incubated for 24h, then treated with drugs.

### Transfection for siRNA

Cells from a fresh culture growing in log phase as described above, and 24h after plating transfected. Prior to transfection, the medium was aspirated and serum-free medium was added to each plate. For transfection, 10 nM of the annealed siRNA, the positive sense control doubled stranded siRNA targeting GAPDH or the negative control (a “scrambled” sequence with no significant homology to any known gene sequences from mouse, rat or human cell lines) were used. Ten nM siRNA (scrambled or experimental) was diluted in serum-free media. Four μl Hiperfect (Qiagen) was added to this mixture and the solution was mixed by pipetting up and down several times. This solution was incubated at room temp for 10 min, then added drop-wise to each dish. The medium in each dish was swirled gently to mix, then incubated at 37 °C for 2h. Serum-containing medium was added to each plate, and cells were incubated at 37 °C for 24h before then treated with drugs (0-24h). Additional immuno-fluorescence/live-dead analyses were performed at the indicated time points.

### Animal studies

Studies were performed according to USDA regulations under VCU IACUC protocol AD20008. B16 mouse melanoma cells (1 × 10^6^) were implanted into the rear flanks of C57 male black mice and tumors permitted to form for 6 days until the mean tumor volume was ∼23 mm^3^. Animals were then segregated into groups with near identical mean volumes and the animals then treated for three days with the indicated therapeutic agents: vehicle control (cremophore); AR42 15 mg/kg (Days 1 and 3); pazopanib 25 mg/kg (Days 1, 2, 3); sodium valproate 50 mg/kg (Days 1, 2, 3) or in combination. Two days after cessation of drug exposure animals are injected IP with: a control IgG (100 μg); an anti-PD-1 IgG (100 μg); or an anti-CTLA4 IgG (100 μg). Tumor volumes were measured prior to drug administration and every three days after the initiation of therapeutic interventions. (n = 10 mice per group +/-SEM). Before, during and after drug treatment tumors are calipered as indicated in the Figure and tumor volume was assessed up to 20-40 days later. When the volume of the tumor reached >1,500 mm^3^, (equivalent of a 4.5 kg tumor in a 60 kg person), animals were humanely sacrificed and the tumor and blood removed for further studies.

### Detection of cell viability, protein expression and protein phosphorylation by immuno-fluorescence using a Hermes WiScan machine

https://www.idea-bio.com/page-87-__Hermes.aspx, Cells (4 × 10^3^) are plated into each well of a 96 well plate, and cells permitted to attach and grow for the next 18h. Based on the experiment, after 18h, cells are then either genetically manipulated, or are treated with drugs. For genetic manipulation, cells are transfected with plasmids or siRNA molecules and incubated for an additional 24h. Cells are treated with vehicle control or with drugs at the indicated final concentrations, alone or in combination. Cells are then isolated for processing at various times following drug exposure. The 96 well plate is centrifuged/cyto-spun to associate dead cells (for live-dead assays) with the base of each well. For live dead assays, after centrifugation, the media is removed and cells treated with live-dead reagent (Thermo Fisher Scientific, Waltham MA) and after 10 min this is removed and the cells in each well are visualized in the Hermes instrument at 10X magnification. Green cells =viable; yellow/red cells = dying/dead. The numbers of viable and dead cells were counted manually from three images taken from each well combined with data from another two wells of separately treated cells (i.e. the data is the mean cell dead from 9 data points from three separate exposures). For immuno-fluorescence studies, after centrifugation, the media is removed and cells are fixed in place and permeabilized using ice cold PBS containing 0.4% paraformaldehyde and 0.5% Triton X-100. After 30 min the cells are washed three times with ice cold PBS and cells are pre-blocked with rat serum for 3h. Cells are then incubated with a primary antibody to detect the expression/phosphorylation of a protein (usually at 1:100 dilution from a commercial vendor) overnight at 37°C. Cells are washed three times with PBS followed by application of the secondary antibody containing an associated fluorescent red or green chemical tag. After 3h of incubation the antibody is removed and the cells washed again. The cells are visualized at either 10X or 60X in the Hermes machine for imaging assessments. All immunofluorescent images for each individual protein/phospho-protein are taken using the identical machine settings so that the levels of signal in each image can be directly compared to the level of signal in the cells treated with drugs. Similarly, for presentation, the enhancement of image brightness/contrast using PhotoShop CS6 is simultaneously performed for each individual set of protein/phospho-protein to permit direct comparison of the image intensity between treatments. Antibodies used include: HSP90 (E289) (Cell Signaling); HSP90 (#2928) (Abcam); HSP90 (ab195575) Abcam; HSP90 3G3 (13495) (Abcam); GRP78 (50b12) (31772) (Cell Signaling); GRP78 (ab191023) Abcam; GRP78 (ab103336) Abcam; GRP78 (N-20) (sc-1050) Santa Cruz; HSP27 (G31) (2402P) Cell Signaling); HSP27 [EP1724Y] (ab62339) Abcam; HSP27 (H-77) (sc-9012) Santa Cruz; HSP27 (LS-C31836) Lifespan science Corp. Other antibodies were as used in prior studies by the laboratory. All immunofluorescent images were initially visualized at 75 dpi using an Odyssey infrared imager (Li-Cor, Lincoln, NE), then processed at 9999 dpi using Adobe Photoshop CS6. For presentation, immunoblots were digitally assessed using the provided Odyssey imager software. Images have their color removed and labeled figures generated in Microsoft PowerPoint.

### Assessment of autophagy

Cells were transfected with a plasmid to express a green fluorescent protein (GFP) and red fluorescent protein (RFP) tagged form of LC3 (ATG8). For analysis of cells transfected with the GFP-RFP-LC3 construct, the GFP/RFP-positive vesicularized cells were examined under the ×40 objective of a Zeiss Axiovert fluorescent microscope.

### Multiplex assays

Were performed as previously described [4]: bio-plex-pro-human-cancer-biomarker-panel-1-16-plex; bio-plex-pro-human-cancer-biomarker-panel-2-18-plex; bio-plex-pro-human-inflammation-panel-1-37-plex; bio-plex-pro-cell-signaling-mapk-panel-9-plex; bio-plex-pro-cell-signaling-akt-panel-8-plex; bio-plex-pro-phospho-egfr-tyr1068-set; bio-plex-pro-phospho-egfr-tyr1173-set; bio-plex-pro-phospho-her-2-tyr1248-set; bio-plex-pro-phospho-ikappab-alpha-ser32-ser36-set; bio-plex-pro-phospho-nf-kappab-p65-ser536-set; bio-plex-pro-phospho-p53-ser15-set; bio-plex-pro-phospho-pdgfr-alpha-tyr754-set; bio-plex-pro-phospho-pdgfr-beta-tyr751-set; bio-plex-pro-phospho-src-tyr416-set; bio-plex-pro-phospho-stat1-tyr701-set; bio-plex-pro-phospho-stat3-tyr705-set; bio-plex-pro-total-akt-set; bio-plex-pro-total-erk1-2-set; bio-plex-pro-total-pten-set; bio-plex-pro-total-ikappab-alpha-set.

### Data analysis

Comparison of the effects of various treatments (performed in triplicate three times) was using one-way analysis of variance and a two tailed Student’s *t*-test. Statistical examination of *in vivo* animal survival data utilized both a two tailed Student’s *t*-test and log rank statistical analyses between the different treatment groups. Differences with a *p*-value of < 0.05 were considered statistically significant. Experiments shown are the means of multiple individual points from multiple experiments (± SEM).

## SUPPLEMENTARY MATERIALS FIGURES



## Abbreviations

ERK: extracellular regulated kinase
PI3K: phosphatidyl inositol 3 kinase
ca: constitutively active
dn: dominant negative
ER: endoplasmic reticulum
mTOR: mammalian target of rapamycin
JAK: Janus Kinase
STAT: Signal Transducers and Activators of Transcription
MAPK: mitogen activated protein kinase
PTEN: phosphatase and tensin homologue on chromosome ten
ROS: reactive oxygen species
CMV: empty vector plasmid or virus
si: small interfering
SCR: scrambled
IP: immunoprecipitation
VEH: vehicle
PAZ: pazopanib
HDAC: histone deacetylase
