# Supplementary material for: HDAC inhibitors enhance the immunotherapy response of melanoma cells
Source: Oncotarget. 2017 May 17;8(47):83155–70. doi: 10.18632/oncotarget.17950 (PMC5669957; doi:10.18632/oncotarget.17950)
Supplement: Supplementary file 1 [file oncotarget-08-83155-s001.pdf]

HDAC inhibitors enhance the immunotherapy response of melanoma cells

SUPPLEMENTARY FIGURES

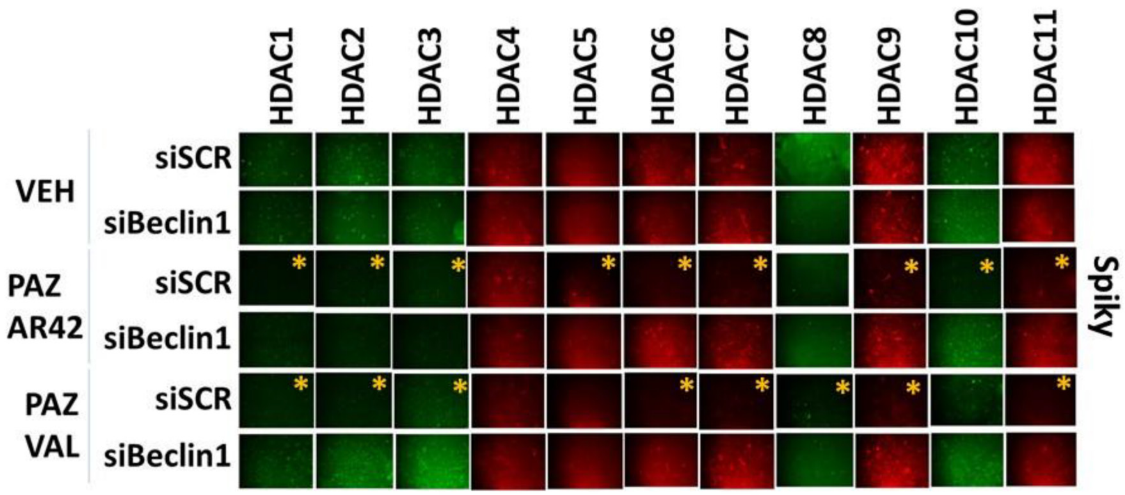

Supplementary Figure 1: Exposure of ovarian carcinoma cells to [pazopanib + HDAC inhibitor] reduces the protein levels of HDACs in a Beclin1-dependent fashion. Spiky ovarian cancer cells were transfected with a scrambled control or with an siRNA to knock down Beclin1. Twenty-four h after transfection cells were treated with vehicle control or with [pazopanib (1 µM) + AR42 (600 nM)] or with [pazopanib (1 µM) + valproate (250 µM)] for 6h. Cells were fixed in place and immunostaining performed to detect the expression of HDACs1-11. * $p < 0.05$ less than corresponding value in vehicle control treated cells.

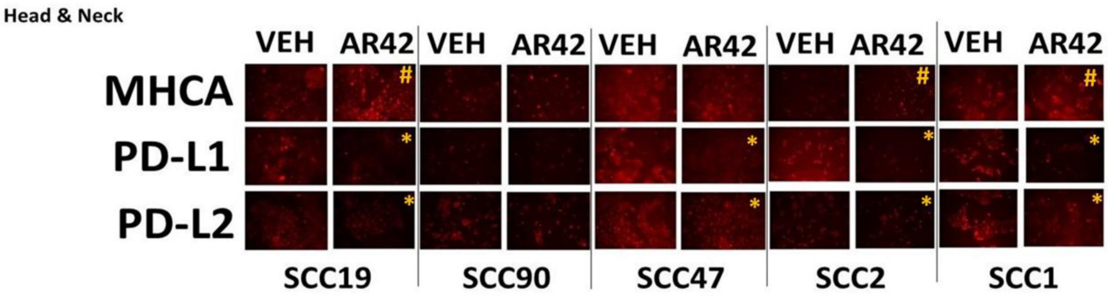

Supplementary Figure 2: AR42 reduces the expression of PD-L1, PD-L2 and ODC in head & neck cancer cells and increases the expression of MHCA. Head & Neck cancer cells were treated with vehicle control or with AR42 (600 nM) for 12h. Cells were fixed in place and immunostaining performed to determine the expression of PD-L1, PD-L2, MHCA and ODC. (n = 3 +/-SEM). * $p < 0.05$ less than vehicle control; # $p < 0.05$ greater than vehicle control.

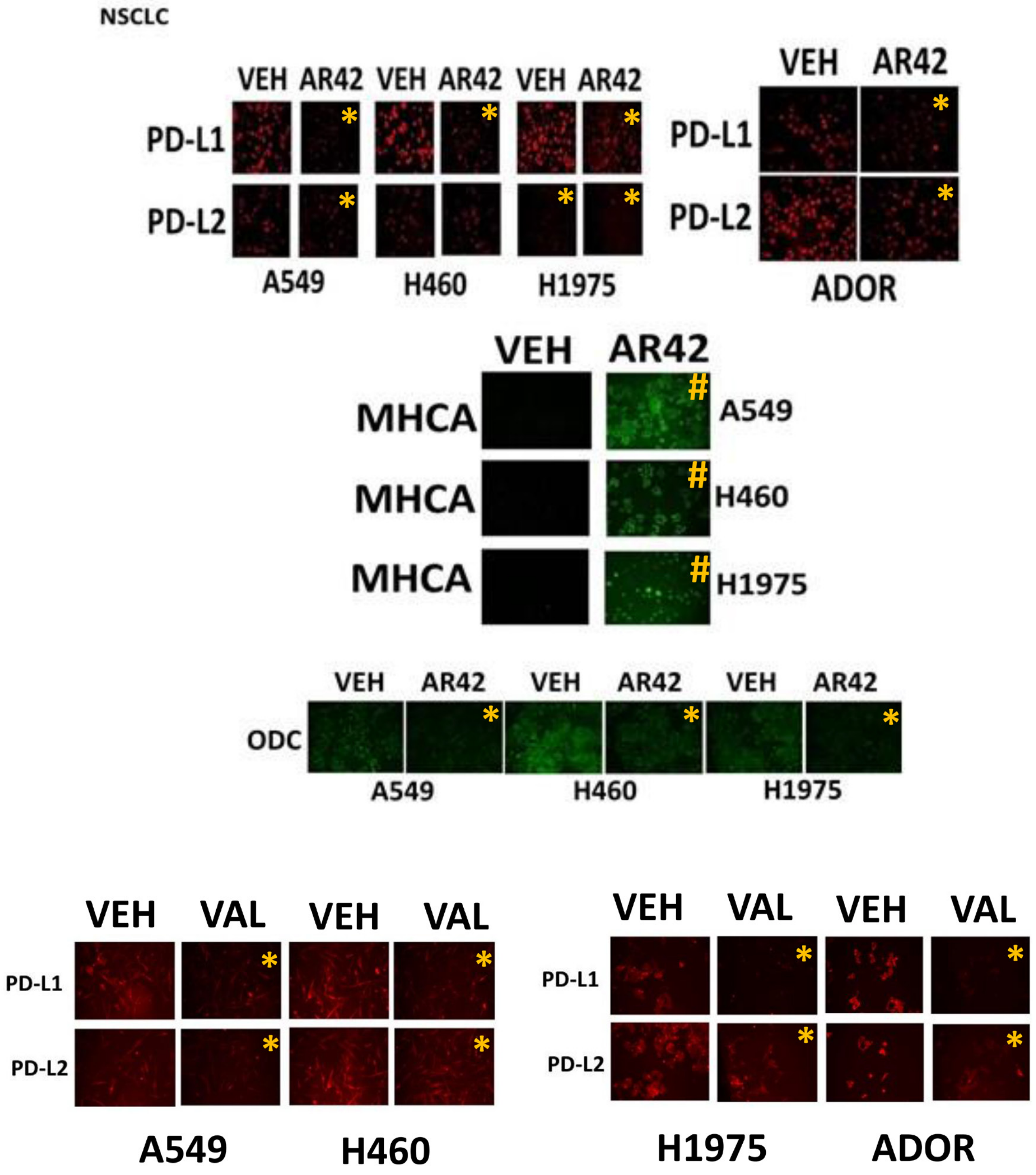

Supplementary Figure 3: AR42 reduces the expression of PD-L1, PD-L2 and ODC in NSCLC cells and increases the expression of MHCA. NSCLC cells were treated with vehicle control or with AR42 (600 nM) or sodium valproate (250 µM), as indicated, for 12h. Cells were fixed in place and immunostaining performed to determine the expression of PD-L1, PD-L2, MHCA and ODC. (n = 3 +/-SEM). * $p < 0.05$ less than vehicle control; # $p < 0.05$ greater than vehicle control.

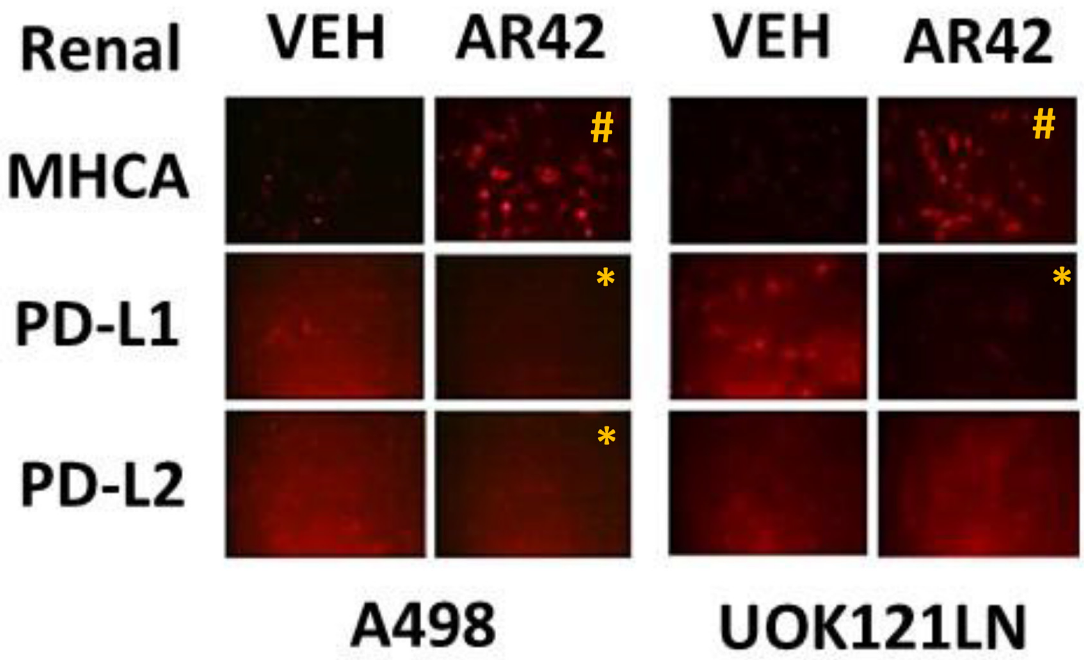

Supplementary Figure 4: AR42 reduces the expression of PD-L1, PD-L2 and ODC in renal cancer cells and increases the expression of MHCA. Renal cancer cells were treated with vehicle control or with AR42 (600 nM) or sodium valproate (250 µM), as indicated, for 12h. Cells were fixed in place and immunostaining performed to determine the expression of PD-L1, PD-L2, MHCA and ODC. (n = 3 +/-SEM). * $p < 0.05$ less than vehicle control; # $p < 0.05$ greater than vehicle control.

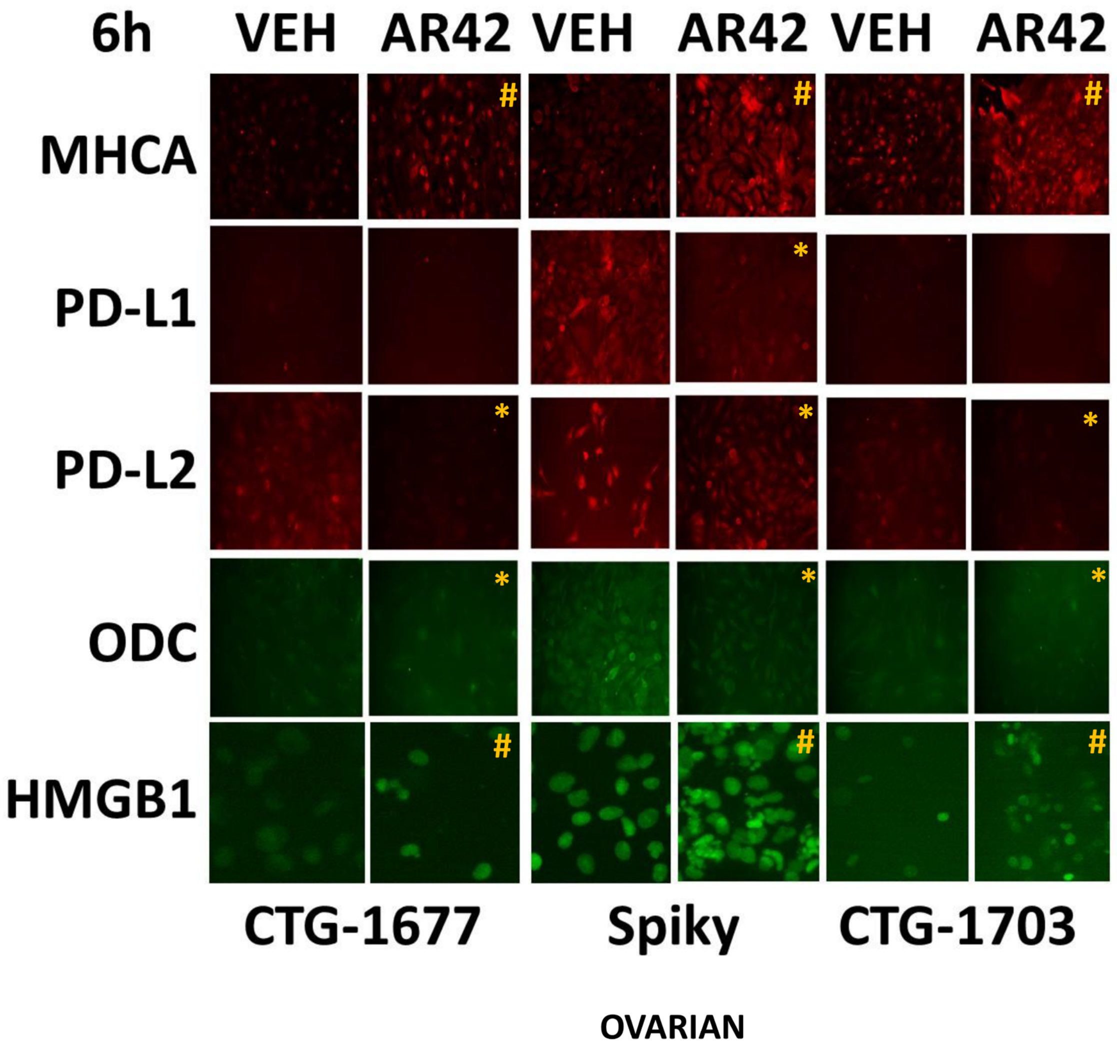

Supplementary Figure 5: AR42 reduces the expression of PD-L1, PD-L2 and ODC in ovarian cancer cells and increases the expression of MHCA. Ovarian cancer cells were treated with vehicle control or with AR42 (600 nM) for 12h. Cells were fixed in place and immunostaining performed to determine the expression of PD-L1, PD-L2, MHCA and ODC, and the localization of HMGB1. (n = 3 +/-SEM). * $p < 0.05$ less than vehicle control; # $p < 0.05$ greater than vehicle control.

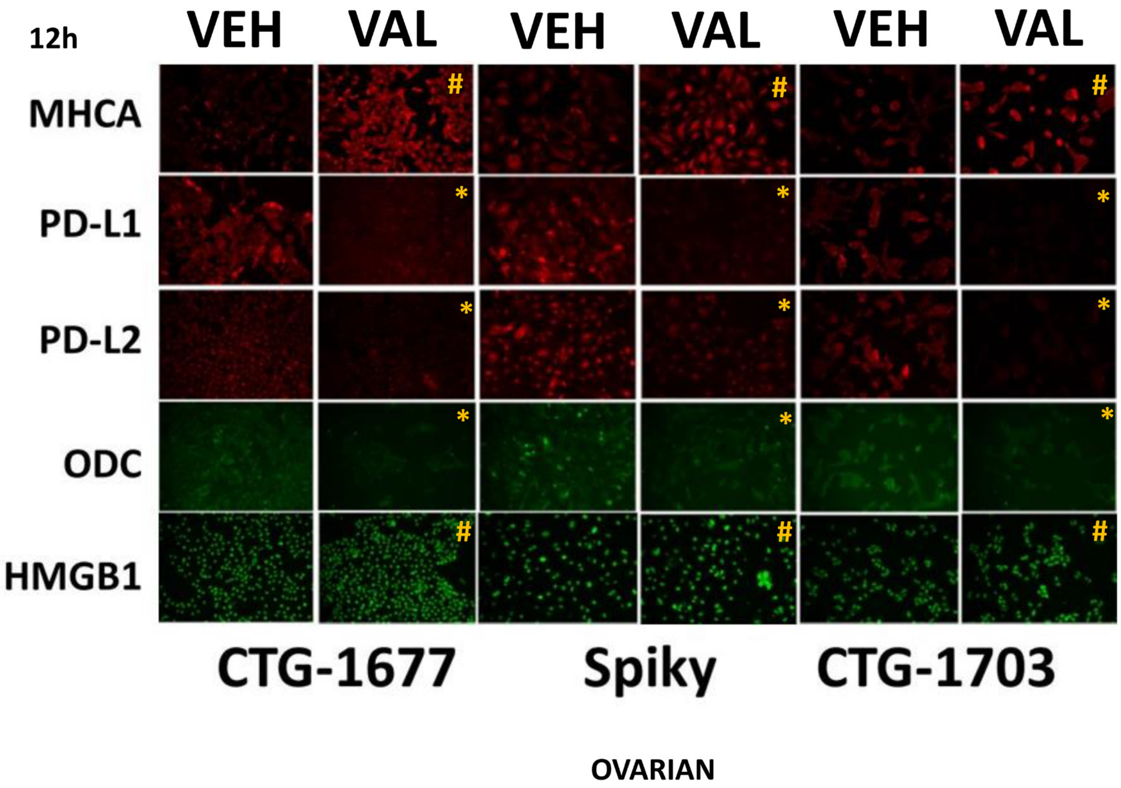

Supplementary Figure 6: Sodium valproate reduces the expression of PD-L1, PD-L2 and ODC in ovarian cancer cells and increases the expression of MHCA. Ovarian cancer cells were treated with vehicle control or with sodium valproate (250 µM) for 12h. Cells were fixed in place and immunostaining performed to determine the expression of PD-L1, PD-L2, MHCA and ODC, and the localization of HMGB1. (n = 3 +/-SEM). * $p < 0.05$ less than vehicle control; # $p < 0.05$ greater than vehicle control.

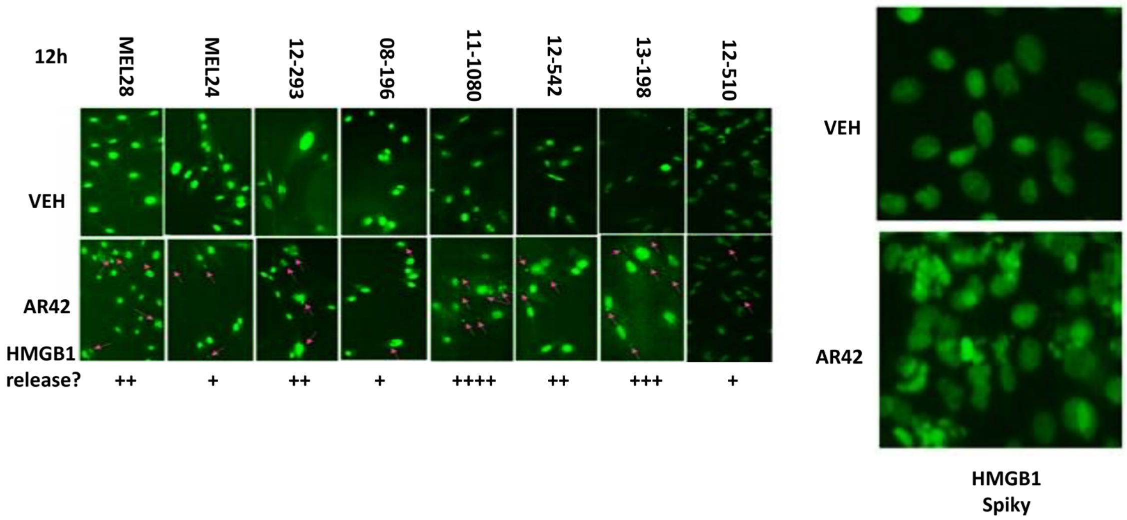

Supplementary Figure 7: AR42 promotes the release of vesicles from cells that stain for HMGB1. Melanoma cells and the ovarian carcinoma PDX isolate Spiky were treated with vehicle control or with AR42 (600 nM). After 12h the cells were fixed in place and immunostaining at 10X magnification performed to determine the expression level and localization of HMGB1. The levels of extracellular HMGB1 vesicle staining were graded as: + (< 10% of cells); ++ (< 20% of cells); +++ (< 40% of cells); ++++ (> 60% of cells).

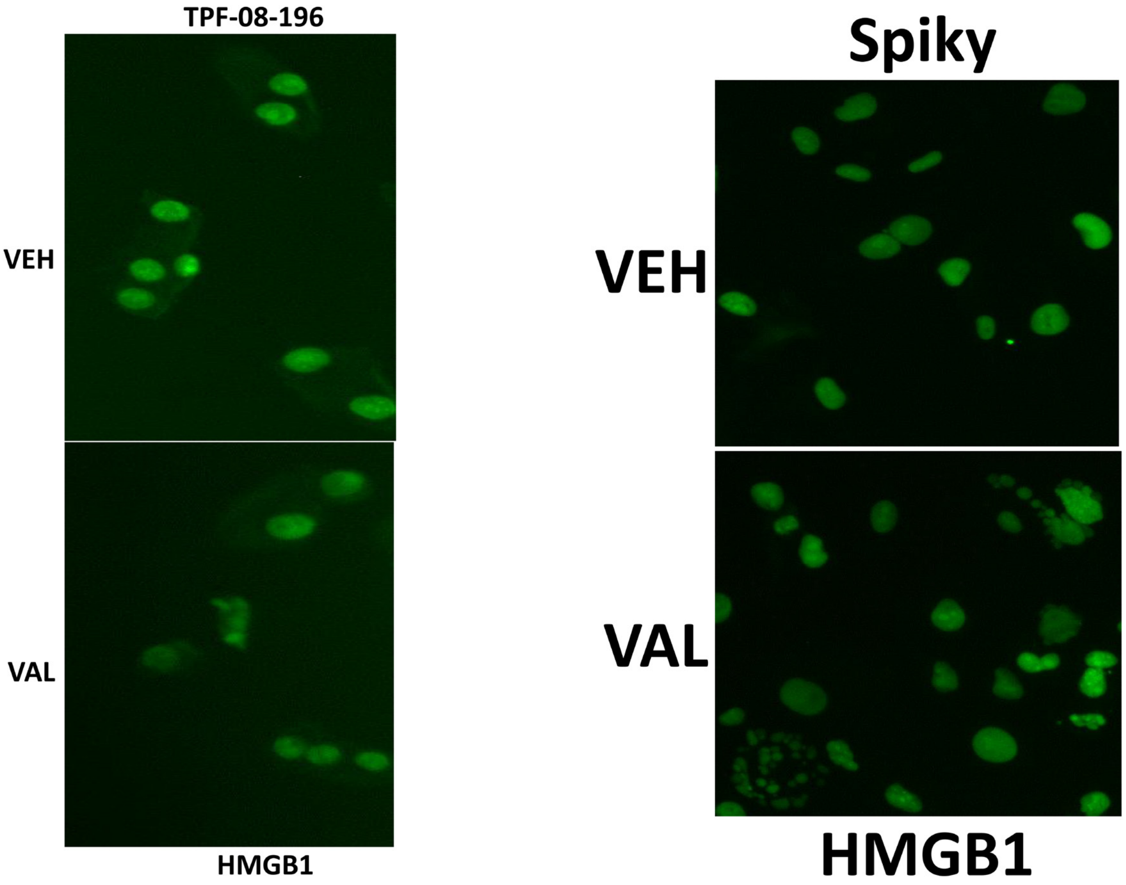

Supplementary Figure 8: Sodium valproate promotes the release of vesicles from cells that stain for HMGB1. Melanoma cells and the ovarian carcinoma PDX isolate Spiky were treated with vehicle control or with sodium valproate (250 μM). After 12h the cells were fixed in place and immunostaining at 10X magnification performed to determine the expression level and localization of HMGB1.

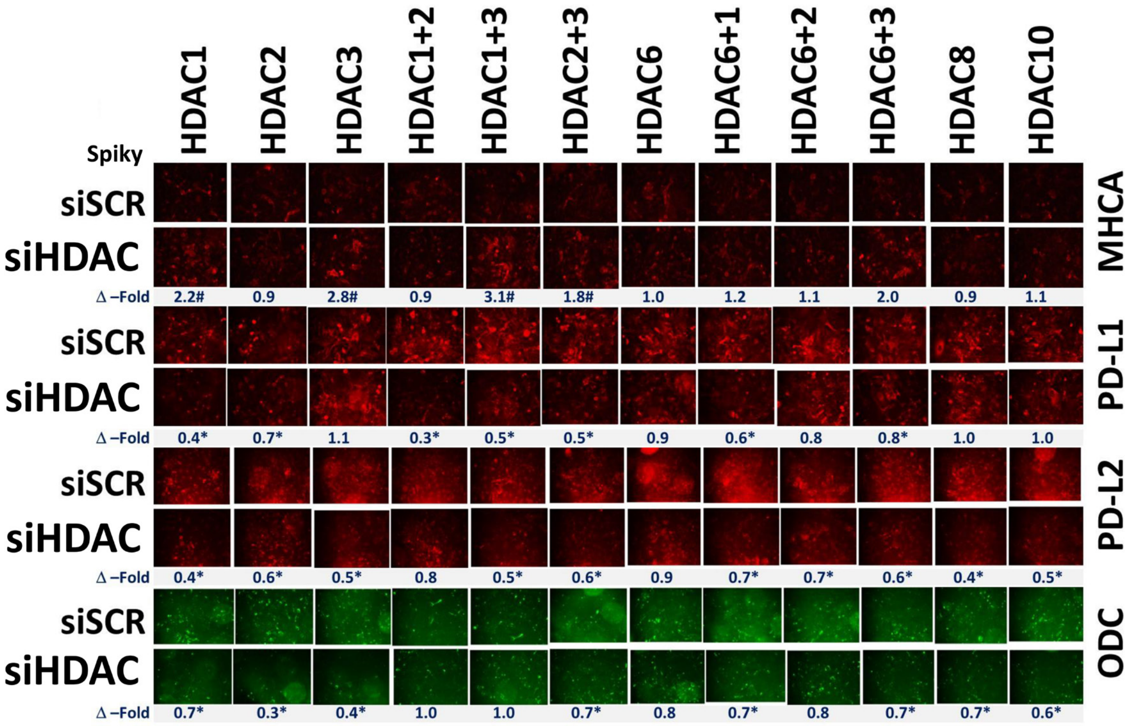

Supplementary Figure 9: Molecular modulation of HDAC expression alters the expression of immune-modulatory markers. Spiky ovarian cancer cells were transfected with a scrambled siRNA (siSCR) or siRNA molecules to knock down: HDAC1, HDAC2, HDAC3, HDAC6, HDAC8 or HDAC10, alone or in the indicated combinations. Twenty-four h after transfection, cells were fixed in place and immunostaining performed to determine the expression levels of PD-L1, PD-L2, MHCA and ODC, and the localization of HMGB1. (n = 3 +/-SEM). * $p < 0.05$ less than siSCR control; # $p < 0.05$ greater than siSCR control.

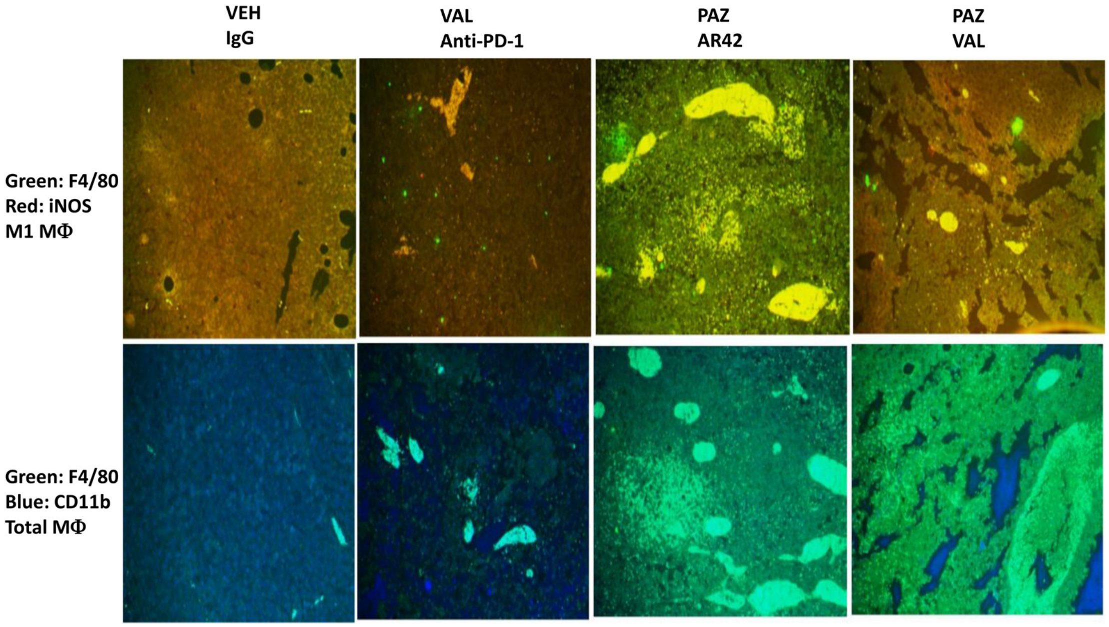

Supplementary Figure 10: Drug treatments increase the levels of M1 macrophages in B16 tumors. Tumors previously treated with the indicated agents were isolated at animal nadir and 5 micron sections made. Sections were stained with the indicated antibodies and visualized at 10X magnification in the Hermes microscope.

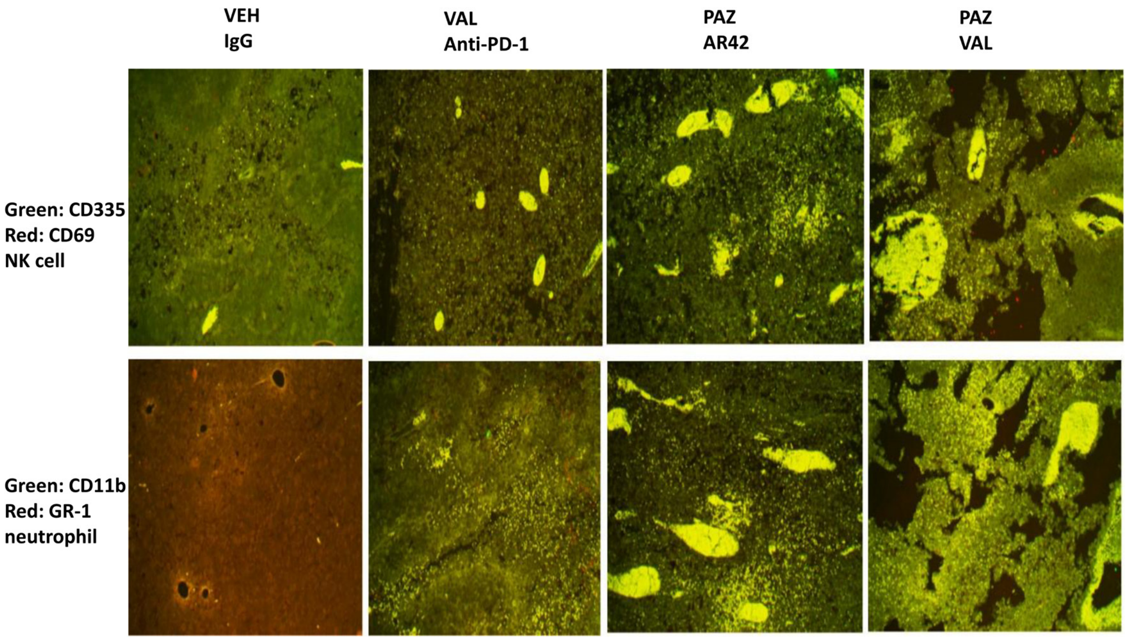

Supplementary Figure 11: Drug treatments increase the numbers of NK cells and neutrophils in B16 tumors. Tumors previously treated with the indicated agents were isolated at animal nadir and 5 micron sections made. Sections were stained with the indicated antibodies and visualized at 10X magnification in the Hermes microscope.

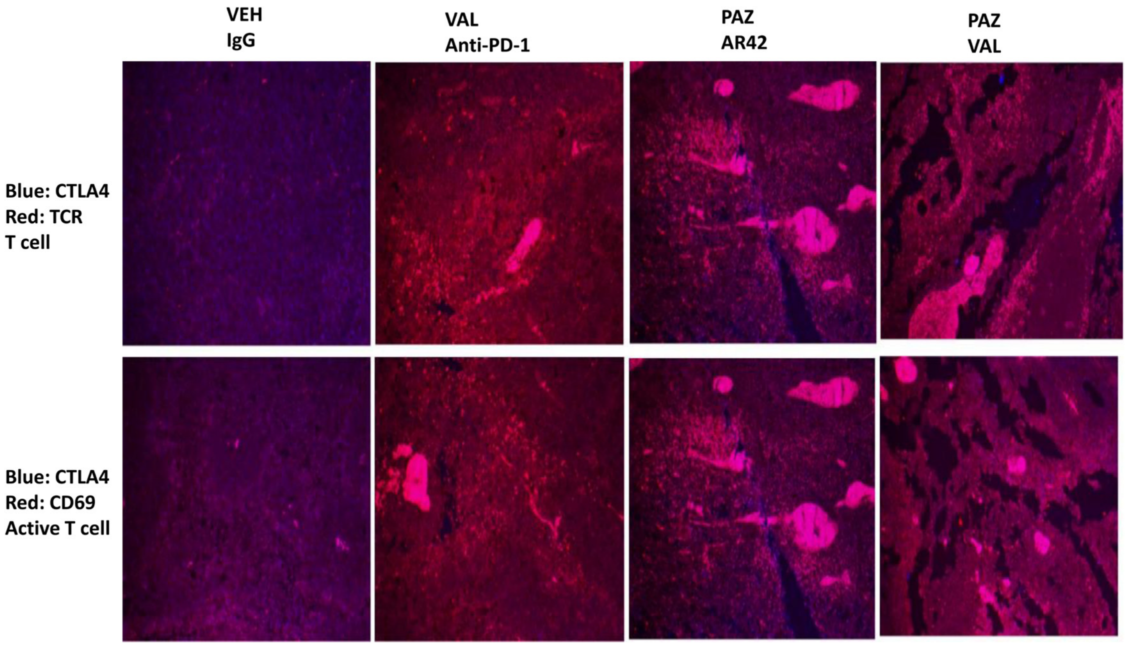

Supplementary Figure 12: Drug treatments increase the total number of T cells and the number of activated T cells in B16 tumors. Tumors previously treated with the indicated agents were isolated at animal nadir and 5 micron sections made. Sections were stained with the indicated antibodies and visualized at 10X magnification in the Hermes microscope.

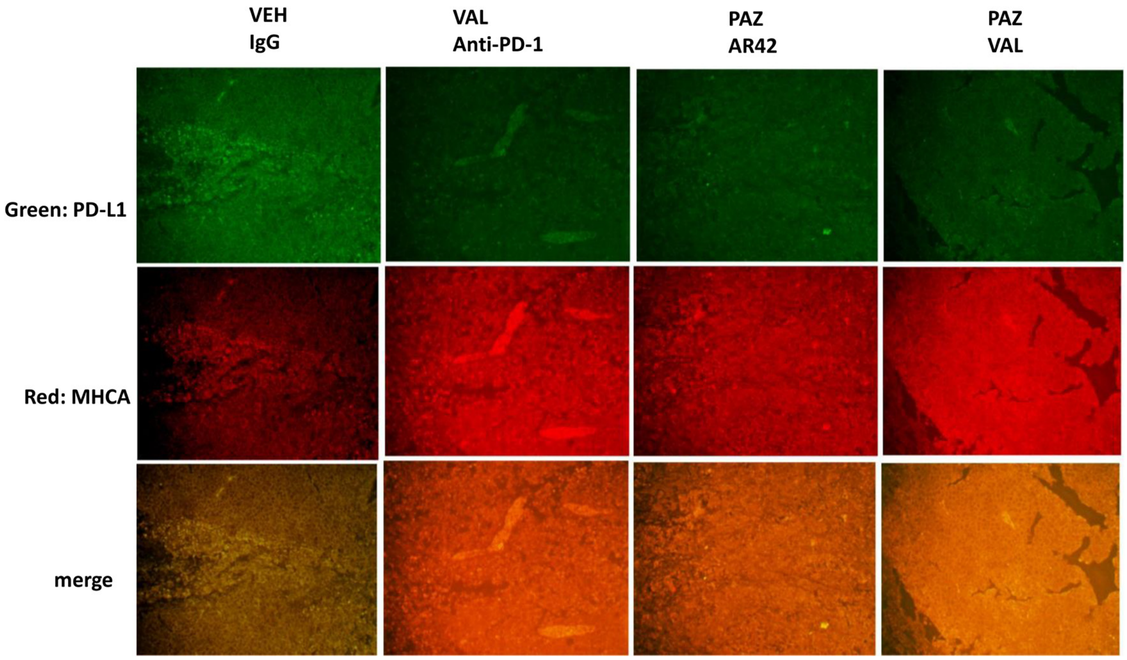

Supplementary Figure 13: Drug treatments increased the levels of MHCA in B16 tumor cells and reduced the levels of PD-L1 on tumor cells and in infiltrating immune cells. Tumors previously treated with the indicated agents were isolated at animal nadir and 5 micron sections made. Sections were stained with the indicated antibodies and visualized at 10X magnification in the Hermes microscope.

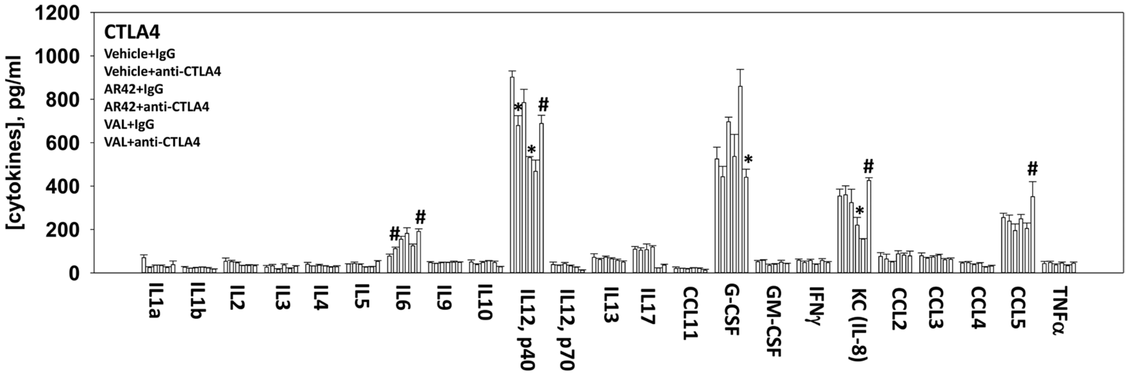

Supplementary Figure 14: Multiplex data comparing vehicle control to HDAC inhibitor in the presence or absence of an anti-CTLA4 antibody (1). Plasma from animals treated with: vehicle control IgG; vehicle control anti-CTLA4; AR42 IgG; AR42 anti-CTLA4; sodium valproate IgG; sodium valproate anti-CTLA4; was processed in the MAGPIX multiplex machine to determine the concentrations of the indicated cytokines (n = 3 separate tumors +/-SEM) * $p < 0.05$ less than vehicle control; # $p < 0.05$ greater than vehicle control.

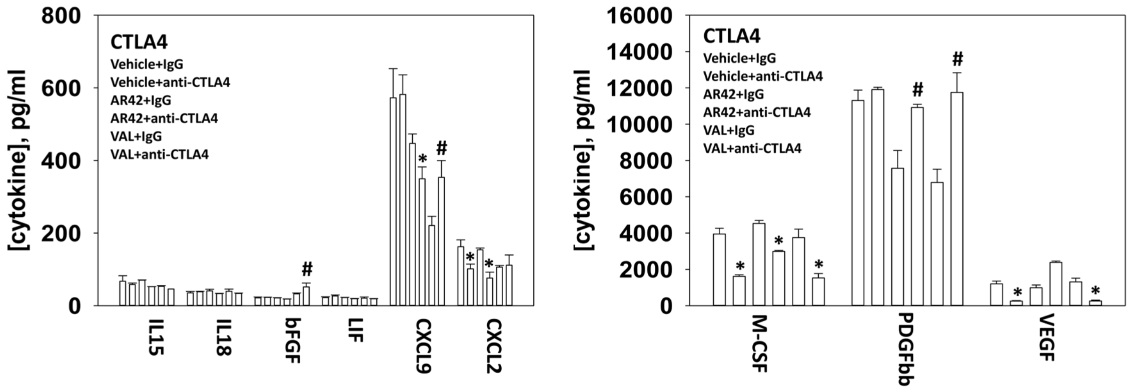

Supplementary Figure 15: Multiplex data comparing vehicle control to HDAC inhibitor in the presence or absence of an anti-CTLA4 antibody (2). Plasma from animals treated with: vehicle control IgG; vehicle control anti-CTLA4; AR42 IgG; AR42 anti-CTLA4; sodium valproate IgG; sodium valproate anti-CTLA4; was processed in the MAGPIX multiplex machine to determine the concentrations of the indicated cytokines (n = 3 separate tumors +/-SEM) * $p < 0.05$ less than vehicle control; # $p < 0.05$ greater than vehicle control.

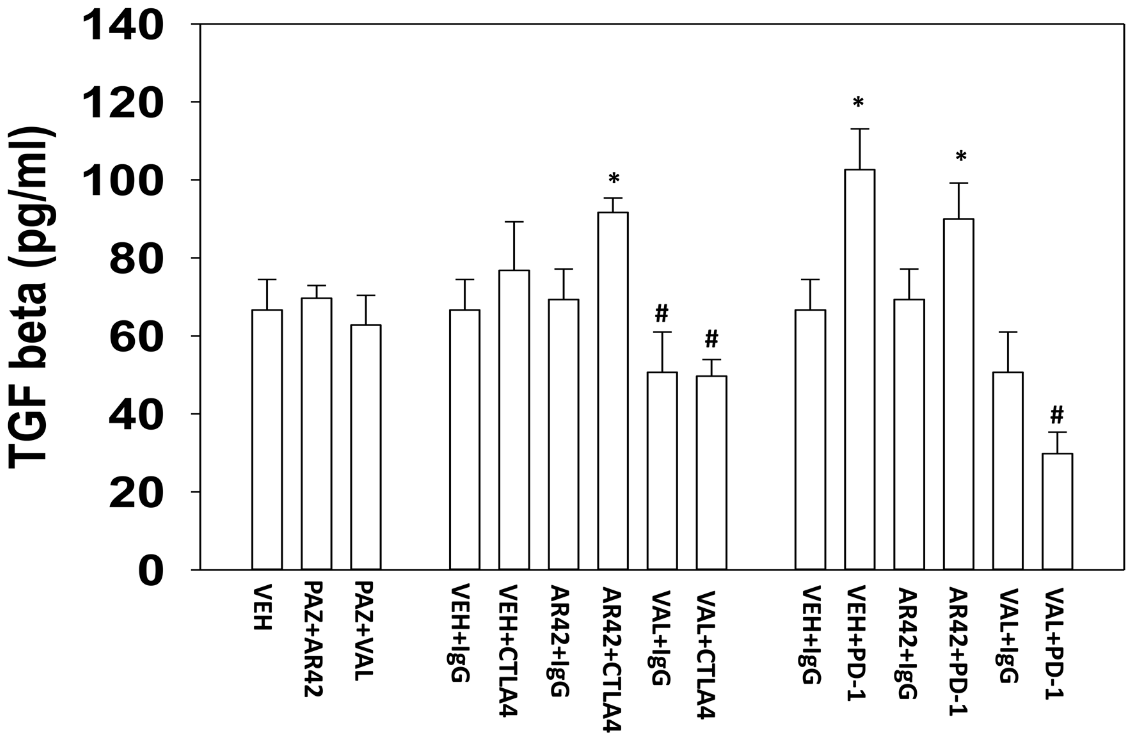

Supplementary Figure 16: Multiplex data comparing vehicle control to HDAC inhibitor in the presence or absence of an anti-CTLA4 antibody (3). Plasma from animals treated with: vehicle control IgG; vehicle control anti-CTLA4; AR42 IgG; AR42 anti-CTLA4; sodium valproate IgG; sodium valproate anti-CTLA4; was processed in the MAGPIX multiplex machine to determine the concentrations of the indicated cytokine TGF beta (n = 3 separate tumors +/-SEM) * $p < 0.05$ less than vehicle control; # $p < 0.05$ greater than vehicle control.

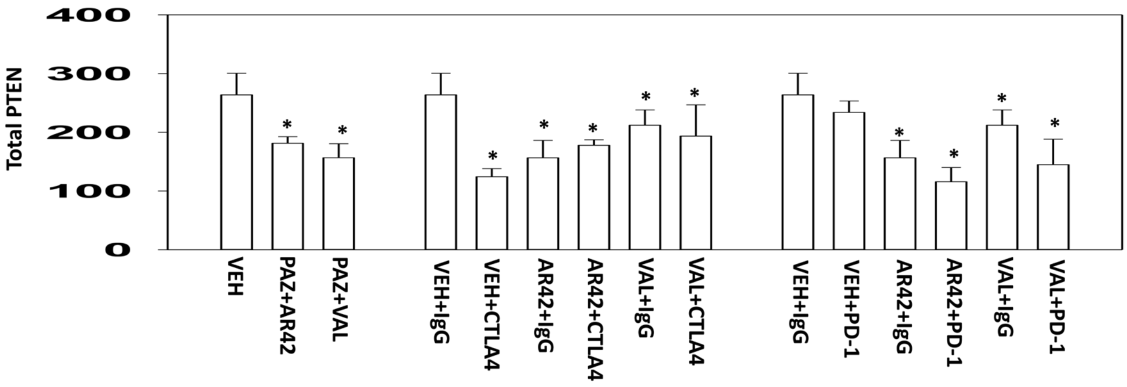

Supplementary Figure 17: Multiplex data comparing vehicle control to HDAC inhibitor in the presence or absence of an anti-CTLA4/anti-PD-1 antibody. Tumor material from animals treated with: vehicle control IgG; vehicle control anti-CTLA4; AR42 IgG; AR42 anti-CTLA4; sodium valproate IgG; sodium valproate anti-CTLA4; was processed in the MAGPIX multiplex machine to determine the total expression of PTEN in the tumor cells (n = 3 separate tumors +/-SEM) * $p < 0.05$ less than vehicle control; # $p < 0.05$ greater than vehicle control.

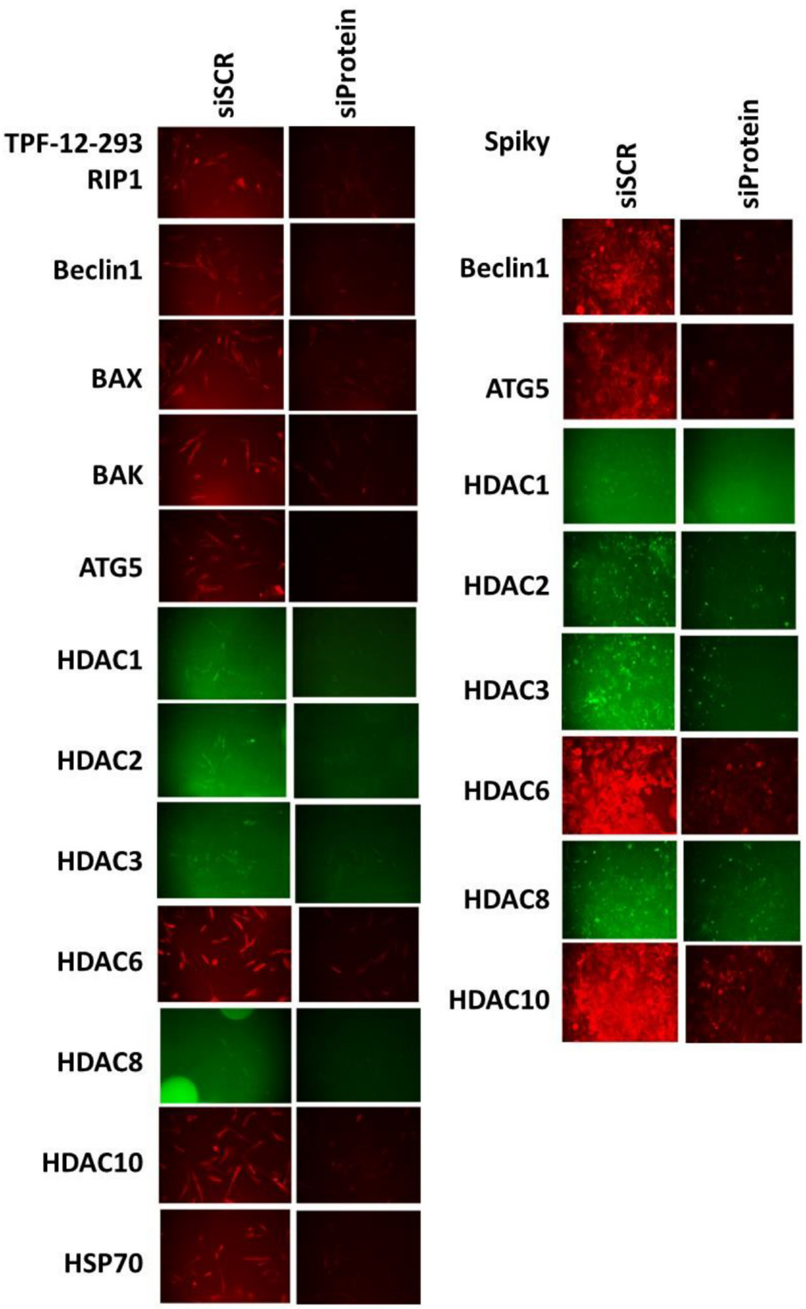

Supplementary Figure 18: Control data sets showing knock down of various proteins after siRNA exposure.
